# Bioactive Phenolics of the Genus *Artemisia* (Asteraceae): HPLC-DAD-ESI-TQ-MS/MS Profile of the Siberian Species and Their Inhibitory Potential Against α-Amylase and α-Glucosidase

**DOI:** 10.3389/fphar.2018.00756

**Published:** 2018-07-12

**Authors:** Daniil N. Olennikov, Nadezhda K. Chirikova, Nina I. Kashchenko, Vyacheslav M. Nikolaev, Sang-Woo Kim, Cecile Vennos

**Affiliations:** ^1^Laboratory of Medical and Biological Research, Institute of General and Experimental Biology, Siberian Division, Russian Academy of Science, Ulan-Ude, Russia; ^2^Department of Biochemistry and Biotechnology, North-Eastern Federal University, Yakutsk, Russia; ^3^Department of Studying the Mechanisms of Adaptation, Scientific Center of Complex Medical Sciences, Yakutsk, Russia; ^4^Department of Biological Sciences, Pusan National University, Busan, South Korea; ^5^Regulatory and Medical Scientific Affairs, Padma AG, Hinwil, Switzerland

**Keywords:** *Artemisia*, Asteraceae, caffeoylquinic acids, flavonoids, high performance liquid chromatography, mass spectrometry, α-amylase inhibition, α-glucosidase inhibition

## Abstract

*Artemisia* genus of Asteraceae family is a source of medicinal plants known worldwide and used as ethnopharmacological remedies for the treatment of diabetes in Northern Asia (Siberia). The aim of this study was to determine the phenolic profile of 12 Siberian *Artemisia* species (*A. anethifolia, A. commutata, A. desertorum, A. integrifolia, A. latifolia, A. leucophylla, A. macrocephala, A. messerschmidtiana, A. palustris, A. sericea, A. tanacetifolia, A. umbrosa*) and to test the efficacy of plant extracts and pure compounds for antidiabetic potential. Finally, by HPLC-DAD-ESI-TQ-MS/MS technique, 112 individual phenolic compounds were detected in *Artemisia* extracts in a wide range of concentrations. Some species accumulated rare plant phenolics, such as coumarin-hemiterpene ethers (lacarol derivatives) from *A. latifolia* and *A. tanacetifolia*; melilotoside from *A. tanacetifolia*; dihydrochalcones (davidigenin analogs) from *A. palustris*; chrysoeriol glucosides from *A. anethifolia, A. sericea*, and *A. umbrosa*; eriodictyol glycosides from *A. messerschmidtiana*; and some uncommon flavones and flavonols. The predominant phenolic group from *Artemisia* species herb was caffeoylquinic acid (CQAs), and in all species, the major CQAs were 5-*O*-CQA (20.28–127.99 μg/g) and 3,5-di-*O*-CQA (7.35–243.61 μg/g). In a series of *in vitro* bioassays, all studied *Artemisia* extracts showed inhibitory activity against principal enzymes of carbohydrate metabolism, such as α-amylase (IC_50_ = 150.24–384.14 μg/mL) and α-glucosidase (IC_50_ = 214.42–754.12 μg/mL). Although many phenolic compounds can be inhibitors, experimental evidence suggests that the CQAs were key to the biological response of *Artemisia* extracts. Mono-, di- and tri-substituted CQAs were assayed and showed inhibition of α-amylase and α-glucosidase, with IC_50_ values of 40.57–172.47 μM and 61.08–1240.35 μM, respectively, and they were more effective than acarbose, a well-known enzyme inhibitor. The results obtained in this study reveal that Siberian *Artemisia* species and CQAs possess a pronounced inhibitory activity against α-amylase and α-glucosidase and could become a complement to synthetic antidiabetic drugs for controlling blood glucose level.

## Introduction

Ethnopharmacology constitutes the scientific basis for the development of active therapeutics based on traditional medicines of various ethnic groups. In recent years, the preservation of local knowledge, the promotion of indigenous medical systems in primary health care and the conservation of biodiversity have become more of a concern to all scientists working at the interface of social and natural sciences. A wide range of innovations in phytochemical analysis allowed an ever-faster analysis and isolation of bioactive natural products (Kumar and Pandey, 2013; Tǎnase et al., 2018) and their identification/structure elucidation (Heinrich, 2010). Novel treatment strategies are needed for all diseases, and recently herbal medicines from such traditions have received attention in the area of prevention or treatment of chronic metabolic disorders, such as diabetes mellitus. Many plant species of various families have been noted for their antidiabetic potential. Among them were known plant extracts that inhibited carbohydrate hydrolysis enzymes, such as α-amylase (Etxeberria et al., 2012; Xiao et al., 2013b) and α-glucosidase (Xiao et al., 2013a), which play a key role in carbohydrate digestion (Yin et al., 2014). The inhibition of these enzymes is one of the possible ways to avert diabetes.

Asteraceae (Compositae) is one of the largest and widespread families of plants, with about 33,000 accepted species. The significance of the Asteraceae family for the curative aims has been described over the centuries. Due to the availability of a large variety of species in this family, it is important in ethnopharmacological medicine throughout the world. *Artemisia* is a large, diverse genus of plants with more than 480 species belonging to Asteraceae. Recent years have witnessed a widespread increase of interest in research of *Artemisia* phytocomponents with antimalarial, cytotoxic, antihepatotoxic, antibacterial and antioxidant activity. (Tan et al., 1998; Bora and Sharma, 2011; Abad et al., 2012; Ivanescu et al., 2015; Pandey and Singh, 2017). It was observed that the various *Artemisia* aqueous and alcoholic extracts possessed an antidiabetic effect caused by hypoglycaemic action (Dabe and Kefale, 2017). Pharmacological data provides convincing evidence that the extracts of *A. absinthium* (Daradka and Abas, 2014), *A. afra* (Afolayan and Sunmonu, 2011), *A. amygdalina* (Ghazanfar et al., 2014), *A. dracundulus* (Ribnicky et al., 2006), *A. judaica* (Nofal et al., 2009), *A. herba-alba* (Awad et al., 2012), *A. ludoviciana* (Anaya-Eugenio et al., 2014), and *A. sphaerocephala* (Xing et al., 2009) were effective in streptozotocin- and alloxan-induced hyperglycemia experimental animal models due to their significant ability to reduce blood glucose level and protect against metabolic aberrations caused by diabetes. The only reported application of *Artemisia* drugs in humans was conducted on type II diabetic individuals and used *A. absinthium* capsules for 30 days (Li et al., 2015). As a result, it was observed that blood glucose level was reduced by 32% compared with the baseline value. This suggests that other *Artemisia* plants may have anti-diabetic properties.

As shown previously by our team, the plants growing at extreme natural habitats, such as Northern Asia or Siberia, are able to accumulate the phenolic components with antidiabetic potential (Olennikov and Kashchenko, 2014, 2015, 2017; Olennikov et al., 2015a, 2017a,b,c; Kashchenko et al., 2017a,b). It is evident that Siberian species of *Artemisia* genus need to be included in our continuing search for the best antidiabetic agents of plant origin.

**Figure 1:**
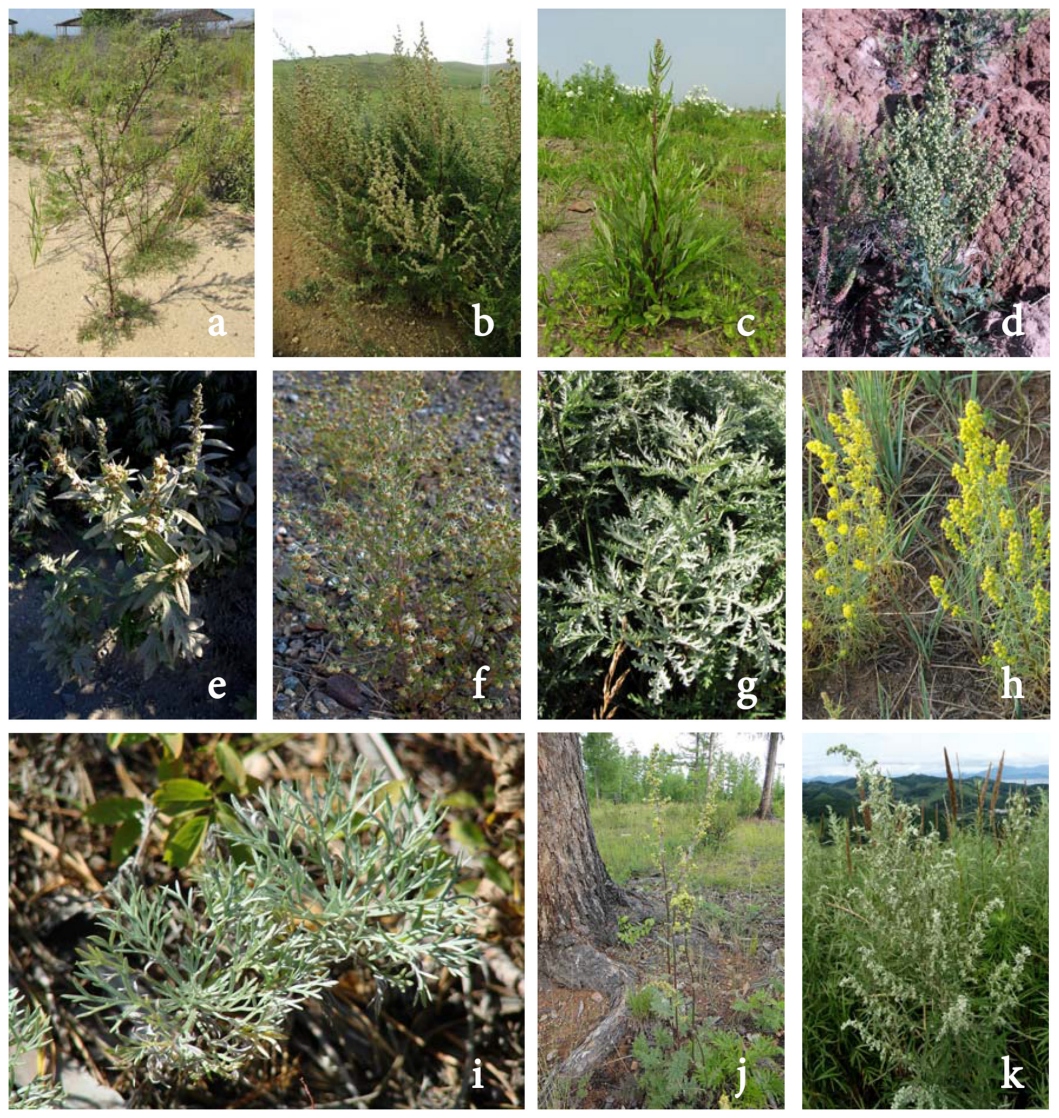
*Artemisia* species mentioned in the present research: *A. commutata* **(a)** *A. desertorum* **(b)** *A. integrifolia* **(c)** *A. latifolia* **(d)** *A. leucophylla* **(e)** *A. macrocephala* **(f)** *A. messerschmidtiana* **(g)** *A. palustris* **(h)** *A. sericea* **(i)** *A. tanacetifolia* **(j)** *A. umbrosa* **(k)**.

Analyzing ethnopharmacological data, at least 12 species of the *Artemisia* genus were noted as used by nomads of Northern Asia or Siberia, such as the ancient Buryat (major northern subgroup of the Mongols) or Yakuts (the largest indigenous Turkic group in Siberia). Doctors of nomads (lamas, healers) applied *Artemisia* plants alone or as components of complex remedies for the prophylaxis and treatment of disorders related to diabetes. These plants were known as *mkhan pa* (*A. commutata, A. integrifolia, A. leucophylla, A. sericea*), *phur nag* or *phur mong* (*A. anethifolia*), *tshar bong* (*A. desertorum, A. latifolia, A. macrocephala, A. messerschmidtiana, A. tanacetifolia, A. umbrosa*), and *yog ma* (*A. palustris*) (Aseeva et al., 2008; Prajnya, 2008; Batorova et al., 2013; Puntsog, 2017; Figure 1).

Even so, none of the mentioned species have been previously examined for their antidiabetic potential. Lastly, with regard to chemical profile of Siberian artemisias, the known data had been very limited. Some terpenoids were detected in *A. anethifolia* (Evstratova and Sinyukhin, 1971; Bakhshyeva et al., 2011); essential oil (Zhu et al., 2013) and flavonoid aglycones (Wang J.-L. et al., 2016) in *A. integrifolia*; sesquiterpenes in *A. latifolia* (Adekenov et al., 1984); essential oil (Shoaib et al., 2015) and sesquiterpenes (Shoaib et al., 2017) in *A. macrocephala*; flavonoids in *A. palustris* (Chemesova et al., 1978); coumarins in *A. tanacetifolia* (Szabó et al., 1985); and sesquiterpenes (Tashenov et al., 2013) and essential oil (Suleimen et al., 2014) in *A. umbrosa*. There is no chemical information about *A. commutata, A. desertorum, A. leucophylla, A. messerschmidtiana*, and *A. sericea*. Overall, the phenolic components of the named species remain a largely unexplored group of *Artemisia* constituents.

As a part of our ongoing research of antidiabetic plant constituents, the present work aimed to conduct chromato-mass-spectrometric profiling of 12 Siberian *Artemisia* species, traditionally used in Siberia as antidiabetic herbs, with high performance liquid chromatography with diode array and electrospray triple quadrupole mass-spectrometric detection (HPLC-DAD-ESI-TQ-MS/MS), as well as analysis of plant extracts and pure components for their inhibitory activity against digestive enzymes (α-amylase, α-glucosidase). This multidisciplinary approach is an essential basis for the advancement of bioactive compounds of the *Artemisia* genus for use in tomorrow's medicines.

## Materials and methods

### Chemicals

The following chemicals were purchased from Carbosynth Limited (Berkshire, UK): 3-*O*-coumaroylquinic acid (Cat. No. FC71593, ≥95%); 6-*O*-feruloylglucose (Cat. No. MF11960, ≥95%); isorhamnetin-3-*O*-galactoside (Cat. No. MI106502, ≥95%); luteolin-6,8-di-*C*-glucoside (lucenin-2; Cat. No. FL157201, ≥95%); nepetin (Cat. No. FE146185, ≥95%); scutellarein-7-*O*-glucoside (Cat. No. FS65327, ≥95%); ChemFaces (Wuhan, China): apigenin-6-*C*-glucoside-4″-*O*-glucoside (isosaponarin, Cat. No. CFN90133, ≥98%), apigenin-6-*C*-glucoside-7-*O*-glucoside (saponarin, Cat. No. CFN90134; ≥98%); apigenin-6-*C*-arabinoside-8-*C*-glucoside (isoschaftoside; Cat. No. CFN92029; ≥98%); apigenin-6,8-di-*C*-glucoside (vicenin-2; Cat. No. CFN92031, ≥98%); apigenin-6-*C*-glucoside-8-*C*-arabinoside (schaftoside; Cat. No. CFN90197, ≥98%); chrysoeriol-7-*O*-glucoside (Cat. No. CFN93021, ≥98%); genkwanin (Cat. No. CFN98670, ≥98%); chrysosplenetin (Cat. No. CFN97026, ≥98%); isorhamnetin-3-*O*-glucoside (Cat. No. CFN99757, ≥98%); luteolin-7-*O*-glucuronide (Cat. No. CFN98512, ≥98%); hispidulin (Cat. No. CFN99491, ≥98%); hispidulin-7-*O*-glucoside homoplantaginin; Cat. No. CFN90344, ≥98%); jaceosidin (Cat. No. CFN90386, ≥98%); kaempferol (Cat. No. CFN98838, ≥98%); nepetin-7-*O*-glucoside (nepetrin; Cat. No. CFN90438, ≥98%); quercetin-3-*O*-rutinoside (rutin; Cat. No. CFN99642, ≥98%); scopoletin-7-*O*-glucoside (scopolin; Cat. No. 531-44-2, ≥98%); 2,4,4′-trihydroxydihydrochalcone (Cat. No. CFN89071, ≥98%), quercetin-3-*O*-galactoside (hyperoside; Cat. No. CFN98754, ≥98%); velutin (Cat. No. CFN98290, ≥98%); sinensetin (Cat. No. CFN99599, ≥98%); Extrasynthese (Lyon, France): apigenin (Cat. No. 1102 S, ≥99%); chrysin (Cat. No. 1362 S, ≥99%); chrysoeriol (Cat. No. 1104 S, ≥ 99%); isorhamnetin-3-*O*-rutinoside (narcissin; Cat. No. 1333 S, ≥99%); luteolin (Cat. No. 1125 S, ≥99%); luteolin-7-*O*-glucoside (cynaroside; Cat. No. 1126 S, ≥98%); scutellarein (Cat. No. 1334 S, ≥99%); Phytopanaceya (Moscow, Russia): chrysoeriol-6,8-di-*C*-glucoside (stellarin-2; Cat. No. 1862, ≥94%), chrysoeriol-7-*O*-glucuronide (Cat. No. 0641, ≥95%); Sigma-Aldrich (St. Louis, MO, USA): acarbose (Cat. No. A8980, ≥95%); acetonitrile for HPLC (Cat. No. 34851, ≥99.9%); aluminum chloride (Cat. No. 206911, ≥98%); 4-aminoantipyrine (Cat. No. A4382); α-amylase from *Aspergillus oryzae* (Cat. No. 10065); apigenin-6-*C*-glucoside (isovitexin; Cat. No. 17804, ≥98%); apigenin-7-*O*-glucoside (cosmosiin; Cat. No. 44692, ≥97%); bovine serum albumin (Cat. No. 05470); 4-*O*-caffeoylquinic acid (Cat. No. 65969, ≥98%); 5-*O*-caffeoylquinic acid (neochlorogenic acid; Cat. No. 94419, ≥98%); 1,3-di-*O*-caffeoylquinic acid (Cat. No. D8196, ≥98%); 3,4-di-*O*-caffeoylquinic acid (Cat. No. SMB00224, ≥90%); 3,5-di-*O*-caffeoylquinic acid (Cat. No. SMB00131, ≥95%); 1,5-di-*O*-caffeoylquinic acid (Cat. No. 16917, ≥98%); 4,5-di-*O*-caffeoylquinic acid (Cat. No. SMB00221, ≥85%); cirsiliol (Cat. No. SML0953, ≥95%); dibasic potassium phosphate (Cat. No. 1551128); sodium phosphate dibasic (Cat. No. 795410); eupatilin (Cat. No. SML1689, ≥98%); dimethyl sulfoxide (Cat. No. D1435, ≥99.5%); eupatorin (Cat. No. E4660, ≥97%); eriodictyol (Cat. No. 74565, ≥95%); esculetin (Cat. No. 68923, ≥97.5%); α-glucosidase from *Saccharomyces cerevisiae* (Cat. No. G5003); glucose oxidase from *Aspergillus niger* (Cat. No. G7141); kaempferol-3-*O*-rutinoside (nicotiflorin; Cat. No. 90242, ≥98%); isorhamnetin (Cat. No. 17794, ≥95%); kaempferol-3-*O*-glucoside (astragalin; Cat. No. 68437, ≥90%); luteolin-6-*C*-glucoside (isoorientin; Cat. No. 02187, ≥98%); melilotoside (Cat. No. SMB00121, ≥95%); methanol (Cat. No. 34860, ≥99%); monobasic potassium phosphate (Cat. No. 1551139); 4-nitrophenyl α-D-glucopyranoside (Cat. No. N1377, ≥99%); peroxidase from horseradish (Cat. No. 77332); phenol (Cat. No. P5566, ≥99.5%); quercetin (Cat. No. Q4951, ≥95%); sodium carbonate anhydrous (Cat. No. 791768, ≥99.5%); umbelliferone-7-*O*-glucoside (skimmin; Cat. No. SMB00511, ≥90%); quercetin-3-*O*-glucoside (isoquercitrin; Cat. No. 16654, ≥98%); sakuranetin (Cat. No. 73422, ≥98%); starch (Cat. No. 03967); water deionized (Cat. No. 38796). Quercetin-3-*O*-(2″-rhamnosyl-)glucoside (calendoflavobioside, ≥95%), quercetin-3-*O*-(4″-rhamnosyl-)glucoside (≥95%), and scopoletin-7-*O*-neohesperidoside were isolated previously from *Calendula officinalis* (Olennikov and Kashchenko, 2013; Olennikov et al., 2017c); 3′-hydroxygenkwanin (≥92%), 5,3′-hydroxy-7,4′-dimetoxyflavone (≥90%), 5-desmethylnobiletin (≥95%), 5-desmethylsinensetin (≥94%) form *Ziziphora pamiroalaica* (Olennikov and Akobirshoeva, 2016); luteolin-6-*C*-glucoside-7-*O*-glucoside (≥94%), luteolin-6-*C*-glucoside-8-*C*-xyloside (lucenin-3, ≥92%), luteolin-6-*C*-xyloside-8-*C*-glucoside (lucenin-1, ≥90%) from *Gastrolychnis tristis* (Olennikov, 2018), 6-hydroxyluteolin (≥94%) from *Scutellaria scordiifolia* (Olennikov and Chirikova, 2013).

### Plant material

The samples of *Artemisia* species were collected in the appropriate flowering period in the Siberian Regions (Russian Federation—Buryatia Republic (BR), Sakha (Yakutia) Republic (SR): *Artemisia anethifolia* Weber ex Stechm.—Yakutsk (Yakutskii region, SR, 23.VII.2017, 62°2′9.6568″ N, 129°41′4.1522″ E, voucher specimen No. AAs/ae-07/14-27/0717); *A. commutata* Besser – Kyren (Tunkinskii district, BR, 25.VII.2017, 51°41′15.4501″ N, 102°8′21.8127″ E, voucher specimen No. AAs/ae-07/16-28/0717); *A. desertorum* Spreng. – Verkhnevilyuysk (Verkhnevilyuysky district, SR, 01.VIII.2017, 63°26′41.3387″ N, 120°19′26.7261″ E, voucher specimen No. AAs/ae-07/11-27/0817); *A. integrifolia* Richards. – Berdigestyakh (Gorny district, SR, 24.VII.2018, 62°5′50.2118″ N, 126°41′3.3254″ E, voucher specimen No. AAs/ae-07/18-27/0717); *A. latifolia* Ledeb. – Kyakhta (Kyakhtinsky district, BR, 02.VIII.17, 50°20′58.8231″ N, 106°28′56.9459″ E, voucher specimen No. AAs/ae-07/13-28/0817); *A. leucophylla* (Turcz. ex Besser) Turcz. ex C.B.Clarke – Ust-Barguzin (Barguzinsky district, BR, 03.VIII.2017, 53°24′58.4813″ N, 109°1′45.0859″ E, voucher specimen No. AAs/ae-07/21-28/0817); *A. macrocephala* Jacquem. ex Besser – Mirny (Mirninsky district, SR, 02.VIII.17, 62°32′23.1008″ N, 113°57′16.7372″ E, voucher specimen No. AAs/ae-07/25-27/0817); *A. messerschmidtiana* Besser – Khandyga (Tomponsky district, SR, 30.07.2017, 62°39′36.7192″ N, 135°33′38.1657″ E, voucher specimen No. AAs/ae-07/12-27/0717); *A. palustris* L. – Babushkin (Kabansky district, BR, 21.VII.2018, 51°42′57.7529″ N, 105°52′39.3969″ E, voucher specimen No. AAs/ae-07/09-28/0717); *A. sericea* Weber ex Stechm. – Churapcha (Churapchinsky district, SR, 20.VII.2017, 62°0′17.6204″ N, 132°25′30.2125″ E, voucher specimen AAs/ae-07/31-27/0717); *A. tanacetifolia* L. – Kurumkan (Kurumkansky district, BR, 02.VIII.17, 54°19′17.4945″ N, 110°20′20.2181″ E, voucher specimen No. AAs/ae-07/22-2800817); *A. umbrosa* (Besser) Turcz. – Zhigansk (Zhigansky district, SR, 18.VII.2017, 66°45′11.5770″ N, 123°23′52.7074″ E, voucher specimen No. AAs/ae-07/32-27/0717). Prof. T.A. Aseeva (IGEB SB RAS, Ulan-Ude) determined the species. The samples of *Artemisia* species were dried in a convective drying oven UT-4610 (Ulab, Sankt-Petersburg, Russia) at 40°C (20–24 hs) up to the humidity level 9–12% and stored at 4 °C in the Institute of General and Experimental Biology Plant Repository. The samples were grounded in an analytical mill A11 basic (IKA®-WerkeGmbH & Co.KG, Staufen, Germany) and then sieved using sieving machine ERL-M1 (Zernotekhnika, Moscow, Russia) up to an average particle diameter of 0.5 mm.

### Total extract preparation

For preparation of the total extract accurately-weighed dried and grounded sample of *Artemisia* herb (100 g) was transferred to a conical glass flask (2 L). After that, 1.5 L of 60% ethanol solution was added with stirring and put in an ultrasonic bath. The extraction conditions were 90 min at 45°C, ultrasound power of 100 W, the frequency 35 kHz. The extraction was repeated three times. The obtained extracts were filtered through a cellulose filter and combined. The filtrates were evaporated *in vacuo* at 50°C until dryness with the use of a rotary evaporator. The total extracts were stored at 4°C until further chemical composition analysis and bioactivity assays.

### SPE fractionation of *Artemisia* extracts

The sample of milled *Artemisia* extract (10 g) was dissolved in 20 mL of 70% ethanol, and then added to 100 mL of distilled water and the mixture was filtered under reduced pressure. Polyamide column (15 g) was prepared: primed with 400 mL methanol followed by 800 mL tridistilled water (td-water). An aliquot (100 mL) of *Artemisia* extract was loaded on polyamide column. Sequential elution was done with 300 mL of td-water (dephenolized fraction) and 500 mL of 70% ethanol (flavonoid-enriched fraction). Fractions were concentrated to dryness under reduced pressure and stored at 4°C until further chemical composition analysis and bioactivity assays.

### Chemical composition assays

The total flavonoid content was estimated as rutin equivalents after spectrophotometric procedure after 5% AlCl_3_ addition (Chirikova et al., 2010). The total caffeoylquinic acid content was determined by the colorimetric Arnow method using 3-*O*-caffeoylquinic acid as the standard (Olennikov et al., 2011).

### Enzyme inhibition assays

The α-glucosidase inhibition assay was performed using spectrophotometric method (Olennikov et al., 2017b). α-Glucosidase from *Saccharomyces cerevisiae* was dissolved in phosphate buffer (pH 6.8) containing bovine serum albumin (2 mg/mL) up to 0.5 U/mL concentration. Solution (10 μL) of sample in DMSO–phosphate buffer, pH 6.8 (1:9) at varying concentrations (10, 100, 250, 500, and 1,000 μg/mL) was premixed with 490 μL of phosphate buffer (pH 6.8) and 250 μL 5 mM *p*-nitrophenyl α-d-glucopyranoside. After preincubating at for 5 min, 250 μL α-glucosidase (0.5 U/mL) was added and incubated at for 15 min. The reaction was terminated by the addition of 2,000 μL Na_2_CO_3_ (200 mM). Absorbance was measured at 400 nm. A solution of acarbose (20 mg/mL) was used as a positive control (PC), and water was used as a negative control (NC). The ability to inhibit α-glucosidase was calculated using the following equation:

$$\begin{array}{l} Inhibitory\;activity\;\left(\%\right)=\frac{\left(A_{400}^{NC}-A_{400}^{PC}\right)-\left(A_{400}^{Sample}-A_{400}^{PC}\right)}{A_{400}^{NC}-A_{400}^{PC}}\times 100, \end{array}$$

where *A*_400_^*NC*^ is the absorbance of the negative control (water) at 400 nm, *A*_400_^*PC*^ is the absorbance of the positive control (acarbose) at 400 nm and *A*_400_^*Sample*^ is the absorbance of the sample solution at 400 nm. The IC_50_ value is the effective concentration at which α-glucosidase activity was inhibited by 50%. Values are expressed as mean obtained from five independent experiments.

α-Amylase inhibitory activity was assayed according to a previously published spectrophotometric protocol (Olennikov and Kashchenko, 2014). Sample solution in DMSO (10 μL) at varying concentrations (10, 100, 250, 500, and 1,000 μg/mL), 30 μL of phosphate buffer (pH 5.0) and 10 μL of α-amylase from *Aspergillus oryzae* (3 U/mL) were incubated for 20 min at . Then 10 μL of 2% starch solution, 40 μL of phosphate buffer (pH 5.0) and 100 μL of the reagent were added and incubated for 30 min at . The reagent was a solution of K_2_HPO_4_ (0.8 mM), KH_2_PO_4_ (0.4 mM), phenol (220 mM), 4-aminoantipyrine (1.5 μM), glucose oxidase from *Aspergillus oryzae* (3 U/mL), and peroxidase from horseradish (0.3 U/mL) in deionized water. Absorbance was measured at 510 nm. A solution of acarbose (20 mg/mL) was used as a positive control (PC), and water was used as a negative control (NC). The ability to inhibit α-amylase was calculated using the following equation:

$$\begin{array}{l} Inhibitory\;activity\;\left(\%\right)=\frac{\left(A_{510}^{NC}-A_{510}^{PC}\right)-\left(A_{510}^{Sample}-A_{510}^{PC}\right)}{A_{510}^{NC}-A_{510}^{PC}}\times 100, \end{array}$$

where *A*_510_^*NC*^ is the absorbance of the negative control (water) at 510 nm, *A*_510_^*PC*^ is the absorbance of the positive control (acarbose) at 510 nm and *A*_510_^*Sample*^ is the absorbance of the sample solution at 510 nm. The IC_50_ value is the effective concentration at which amylase activity was inhibited by 50%. Values are expressed as mean obtained from five independent experiments.

### HPLC-DAD-ESI-TQ-MS/MS profiling and compounds identification condition

Reversed-phase high-performance liquid chromatography with diode array detection and electrospray ionization mass spectrometry (RP-HPLC-DAD-ESI-TQ-MS/MS) procedure was used for the phenolic compounds profiling. Experiments were performed on an LCMS 8050 liquid chromatograph coupled with diode-array-detector and triple-quadrupole electrospray ionization detector (Shimadzu, Columbia, MD, USA), using a ProntoSIL-120-5-C18 AQ column (1 × 50 mm, Ø 1 μm; Metrohm AG; Herisau, Switzerland), column temperature was . Eluent A was water and eluent B was acetonitrile. The injection volume was 1 μL, and elution flow was 100 μL/min. Gradient program: 0.0–1.0 min 5–21% B, 1.0–2.0 min 21–38% B, 2.0–2.7 min 38–55% B, 2.7–3.5 min 55–61% B, 3.5–5.0 min 61–94% B. The DAD acquisitions were performed in the range of 200–600 nm and chromatograms were integrated at 280 nm. For ESI-MS, the parameters were set as follows: temperature levels of ESI interface, desolvation line and heat block were 300, 250, and 400°C, respectively; the flow levels of nebulizing gas (N_2_), heating gas (air) and collision-induced dissociation gas (Ar) were 3, 10, and 0.3 mL/min, respectively. The capillary voltage was kept at +3 kV (coumarins) in positive mode and at −4.5 kV (phenylpropanoids, dihydrochalcones and flavonoids) in negative mode. ESI-MS spectra were recorded by scanning in the range of *m*/*z* 100–1,900. The identification of compounds was done by analysis of their retention time, ultraviolet and mass-spectrometric data comparing the same parameters with the reference samples and / or literature data. The product ion spectra of the selected precursor ions (MS/MS) were used to improve the reliability of identification of *Artemisia* phenolic compounds.

### HPLC-DAD quantification condition

Quantification of the phenolic compounds was performed in HPLC-DAD experiments using chromatographic conditions mentioned above. To prepare the stock solutions of reference compounds, 15 mg of 4-*O*-caffeoylquinic acid, 5-*O*-caffeoylquinic acid, 1,3-di-*O*-caffeoylquinic acid, 3,4-di-*O*-caffeoylquinic acid, 3,5-di-*O*-caffeoylquinic acid, 4,5-di-*O*-caffeoylquinic acid, apigenin-6-*C*-glucoside-4″-*O*-glucoside (isosaponarin), quercetin-3-*O*-(2″-rhamnosyl-)glucoside (calendoflavobioside), quercetin-3-*O*-(4″-rhamnosyl-)glucoside, quercetin-3-*O*-(6″-rhamnosyl-)glucoside (rutin), quercetin-3-*O*-glucoside (isoquercitrin), quercetin-3-*O*-galactoside (hyperoside), kaempferol-3-O-(6″-rhamnosyl-)glucoside (nicotiflorin), isorhamnetin-3-*O*-glucoside, and quercetin were accurately weighed and individually dissolved in DMSO/methanol mixture (1:4) in volumetric flasks (1 mL). The external standard calibration curve was generated using 10 data points, covering the concentration ranges 1–1,000 μg/mL. The calibration curves were created by plotting the peak area vs. the concentration levels. Scopoletin-7-*O*-neohesperidoside (t_R_ 0.92 min) was used as the internal standards and was dissolved separately in DMSO/methanol mixture (1:4) at concentration 1,000 μg/mL. All the analyses were carried out in triplicate and the data were expressed as mean value ± standard deviation (SD). For preparation of extract solution, an accurately weighed extract of *Artemisia* plant (10 mg) was placed in an Eppendorf tube, 1 mL of 60% ethanol was added, and the mixture was weighted. Then the sample was extracted in an ultrasonic bath for 10 min at 40°C. After cooling, the tube weight was reduced to initial sign, and the resultant extract was filtered through a 0.22-μm PTFE syringe filter before injection into the HPLC system for analysis.

### Method validation

For validation of the analytical method, the guidelines established by the International Conference on the Harmonization of Technical Requirements for the Registration of Pharmaceuticals for Human Use (ICH) were employed (2005). The linearity of the method was studied by injecting five known concentrations of the standard compounds in the defined range. Results from each analysis were averaged and subjected to regression analysis. Limits of detection (LOD) and quantification (LOQ) were determined using the following equations:

$$\begin{array}{l} \text{LOD }=\;\left(3.3\times {\text{S}}_{\text{YX}}\right)/\text{a};\text{LOQ }=\;\left(10\times {\text{S}}_{\text{YX}}\right)/\text{a}, \end{array}$$

where *S*_YX_ is a standard deviation of the response (Y intercept) and *a* is a slope of calibration curve. The precision of the analytical method was evaluated by intra-day, inter-day, and repeatability test. Intra-day assay was determined by assaying the mixture solution containing 15 standards (50 μg/mL) during the same day (five injections), and inter-day assay was analyzed using the same concentration for intra-day precision on four different days (interval of 1 day) in the same laboratory. The repeatability test of the sample was performed on 7-fold experiments of the mixture solution containing 15 standards (100 μg/mL). The stability test was performed with one sample solution, which was stored at room temperature and analyzed at 0, 2, 4, 8, 12, 24, and 48 h. For analysis of recovery data, the appropriate amounts of the powdered sample of 15 standards were weighted and spiked with a known amount of each reference compound and then analyzed. Each sample was analyzed in five times.

### Statistical and multivariative analysis

Statistical analyses were performed using a one-way analysis of variance (ANOVA), and the significance of the mean difference was determined by Duncan's multiple range test. Differences at *p* < 0.05 were considered statistically significant. The results are presented as mean values ± SD (standard deviations) of the three replicates. Advanced Grapher 2.2 (Alentum Software Inc., Ramat-Gan, Israel) was used to perform linear regression analysis and to generate graphs. Principal component analysis (PCA) based on a data matrix (15 markers × 12 samples) was performed using Graphs 2.0 utility for Microsoft Excel (Komi NTc URO RAN, Syktyvkar, Russia) to generate an overview for groups clustering.

## Results

### Phenolic group content and inhibitory activity against α-amylase and α-glucosidase of 12 *Artemisia* species

At the preliminary stage of the investigation, the extracts of *Artemisia* species were obtained and the yields of the extracts were determined (Table 1). The average value of extraction yield of *Artemisia* species was 29.26 %. Further, the characterization of the phenolic group content was performed. There were significant variations in flavonoid and caffeoylquinic acids (CQAs) contents of *Artemisia* species. The maximum flavonoid content was observed in *A. palustris* extract (202.67 mg/g), in turn, minimal value was evident for *A. desertorum* extract (2.46 mg/g). The content of CQAs in *A. commutata* extract (514.65 mg/g) considerably exceeded the values of CQAs for other *Artemisia* species. Minimal CQAs value was detected in *A. sericea* extract (26.46 mg/g).

**Table 1:** Yield of extraction (% of dry plant weight), total flavonoid and caffeoylquinic acids (CQAs) content (mg/g) and inhibitory activity of *Artemisia* extracts against α-amylase and α-glucosidase (IC_50_, μg/mL).

***Artemisia* extract**	**Yield (%)**	**Phenolic group content (mg/g)**		**Inhibitory activity, IC_50_ (μg/mL)**	
		**Flavonoids**	**CQAs**	α**-Amylase**	α**-Glucosidase**
*A. anethifolia*	25.0	56.52 ± 0.93	172.86 ± 4.49	243.82 ± 8.77^c^	401.25 ± 12.84^c^
*A. commutata*	24.5	30.88 ± 0.61	514.65 ± 15.43	150.24 ± 6.15^a^	214.42 ± 6.00^a^
*A. desertorum*	28.2	2.46 ± 0.05	104.78 ± 2.93	365.25 ± 14.24^f^	511.36 ± 16.36^e^
*A. integrifolia*	23.9	15.14 ± 0.30	190.53 ± 5.14	214.78 ± 8.59^bc^	374.90 ± 11.62^bc^
*A. latifolia*	23.2	63.95 ± 1.15	100.53 ± 3.11	355.31 ± 15.27^ef^	568.48 ± 15.92^ef^
*A. leucophylla*	25.8	19.67 ± 0.41	202.63 ± 6.07	215.70 ± 7.33^bc^	325.63 ± 9.44^ab^
*A. macrocephala*	29.5	38.48 ± 0.76	108.68 ± 3.36	274.11 ± 10.69^d^	371.66 ± 12.71^b^
*A. messerschmidtiana*	37.5	11.19 ± 0.23	211.47 ± 3.81	233.93 ± 9.35^c^	354.10 ± 11.33^b^
*A. palustris*	38.4	202.67 ± 4.45	32.06 ± 0.54	268.35 ± 11.00^d^	684.32 ± 21.20^fg^
*A. sericea*	28.3	25.80 ± 0.57	26.46 ± 0.79	384.14 ± 14.59^f^	754.12 ± 21.03^gh^
*A. tanacetifolia*	34.3	14.87 ± 0.31	175.14 ± 4.90	233.94 ± 8.87^c^	408.03 ± 11.42^cd^
*A. umbrosa*	32.5	18.24 ± 0.38	129.46 ± 4.01	207.12 ± 7.62^ab^	444.38 ± 14.78^d^
Acarbose	–	–	–	311.24 ± 8.09^e^	1209.59 ± 7.02^h^

*Mean values ± standard deviations. Statistical analysis was performed by one-way ANOVA (Tukey test). In each column, different letters (a–h) stand for significant statistical different data (p < 0.05)*.

After determination of the phenolic contents, the investigation of α-amylase and α-glucosidase inhibitory activity of the extracts examined was conducted. Enzymatic analysis showed that *A. commutata* extract demonstrated an ability to inhibit α-amylase activity with the highest value (IC_50_ = 150.24 μg/mL) and the lowest value for *A. sericea* extract (IC_50_ = 384.14 μg/L). A similar trend was seen in the α-glucosidase inhibition experiments. *A. commutata* extract was the most active (IC_50_ = 150.24 μg/mL) and *A. sericea* extract displayed considerably worse inhibitor activity of α-glucosidase (IC_50_ = 754.12 μg/mL). The remaining *Artemisia* species showed inhibitory activity when present in concentrations higher than 207.12 μg/mL for α-amylase, and 325.63 μg/mL in the case of α-glucosidase. Acarbose inhibited α-amylase and α-glucosidase with IC_50_ values of 311.24 and 1209.59 μg/mL, respectively.

**Figure 2:**
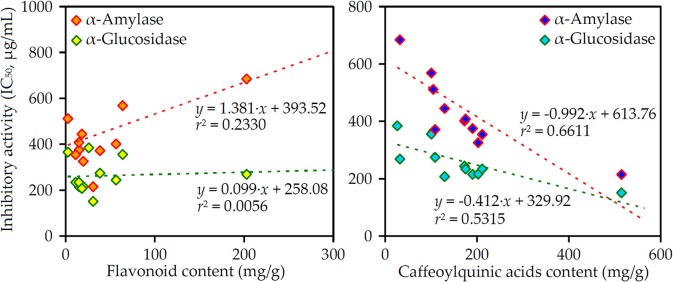
Correlation graphs between flavonoid/caffeoylquinic acids content (mg/g) in the *Artemisia* extracts and their α-amylase/α-glucosidase inhibitory activity (IC_50_, μg/mL).

To understand the links among all the studied chemical parameters and biological potential, linear regression analysis was used (Figure 2). The highest correlation was observed between total CQAs content and enzyme inhibiting activity (*r*^2^ = 0.5315–0.6611) opposite total flavonoid content demonstrating weak relationships (*r*^2^ = 0.0056–0.2330).

Importantly, the elimination of the phenolic compounds from *Artemisia* extracts after SPE procedure caused a drastic reduction of α-amylase and α-glucosidase inhibiting activity of the resultant dephenolized extracts (Supplementary Table S1).

### Phenolic profile of 12 *Artemisia* species by HPLC-DAD-ESI-TQ-MS/MS

**Figure 3:**
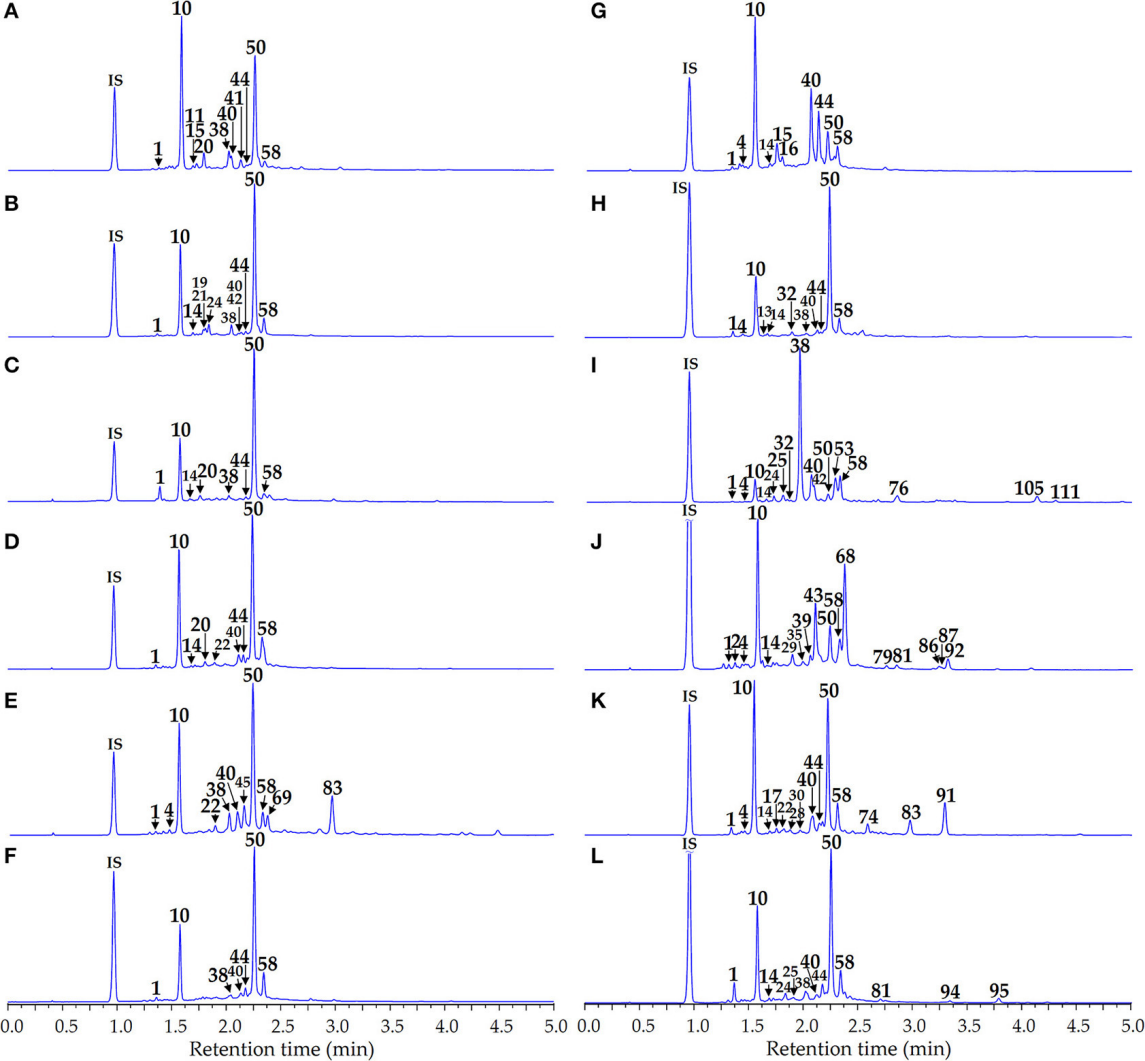
HPLC-DAD chromatograms of the total extracts of *Artemisia* species at 280 nm. **(A)** *A. anethifolia*; **(B)** *A. commutata*; **(C)** *A. desertorum*; **(D)** *A. integrifolia*; **(E)** *A. latifolia*; **(F)** *A. leucophylla*; **(G)** *A. macrocephala*; **(H)** *A. messerschmidtiana*; **(I)** *A. palustris*; **(J)** *A. sericea*; **(K)** *A. tanacetifolia*; **(L)** *A. umbrosa*. Compounds are numbered as listed in Table 2. IS, internal standard (scopoletin-7-*O*-neohesperidoside).

**Figure 4:**
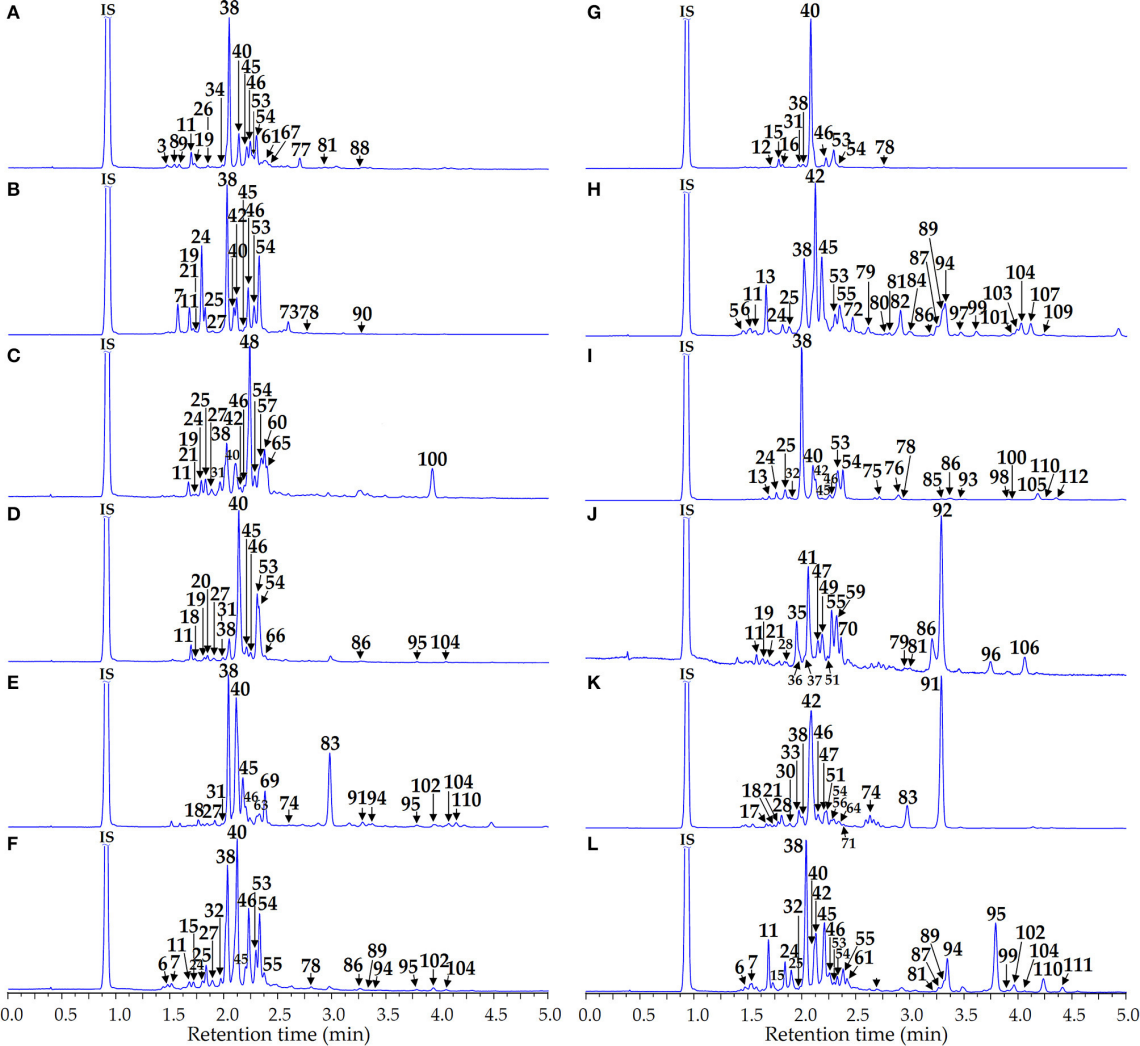
HPLC-DAD chromatograms of the flavonoid-enriched SPE fractions of *Artemisia* extracts at 280 nm. **(A)** *A. anethifolia*; **(B)** *A. commutata*; **(C)** *A. desertorum*; **(D)** *A. integrifolia*; **(E)** *A. latifolia*; **(F)** *A. leucophylla*; **(G)** *A. macrocephala*; **(H)** *A. messerschmidtiana*; **(I)** *A. palustris*; **(J)** *A. sericea*; **(K)** *A. tanacetifolia*; **(L)** *A. umbrosa*. Compounds are numbered as listed in Table 2. IS, internal standard (scopoletin-7-*O*-neohesperidoside).

Phenolome profiling of the *Artemisia* species was implemented after high performance liquid chromatography (HPLC) separation of total methanolic extracts (Figure 3) and flavonoid-enriched SPE fractions (Figure 4) using diode array detection (DAD) and triple quadrupole electrospray ionization detection (TQ-ESI-MS), with both positive and negative mode.

The identification data summarized in Table 2 allowed us to detect a total of 112 phenolic compounds in 12 *Artemisia* extracts, including phenylpropanoid quinates and glycosides, simple phenolic acid, coumarins, dihydrochalcones and flavonoids (flavones, flavonols, flavanones) both in glycosidic and aglycone form.

**Table 2:** Identified compounds in *Artemisia* extracts.

**Peak No**	**Rt (min)**	**Compound**	**UV (nm) λ_max_**	**MS fragments (*m*/*z*)**	***Artemisia* extract**											
					***A. anethifolia***	***A. commutata***	***A. desertorum***	***A. integrifolia***	***A. latifolia***	***A. leucophylla***	***A. macrocephala***	***A. messerschmidtiana***	***A. palustris***	***A. sericea***	***A. tanacetifolia***	***A. umbrosa***
1	1.30	4-*O*-Caffeoylquinic acid^a^	326	353 [M-H]^−^, 191	+	+	+	+	+	+	+	+	+	+	+	+
2	1.31	Protocatechuic acid-*O*-Glc^b^	255, 293	315 [M-H]^−^, 153 [(M-Glc)-H]^−^										+		
3	1.44	Dihydroferulic acid-*O*-Glc^b^	269, 306	357 [M-H]^−^, 195 [(M-Glc)-H]^−^	+											
4	1.46	Umbelliferone-7-*O*-Glc (skimmin)^a^	260, 317	347 [M+Na]^+^, 325 [M+H]^+^, 163 [(M-Glc)+H]^+^					+		+	+	+	+	+	
5	1.47	Caffeic acid-*O*-Glc^b^	325	341 [M-H]^−^, 179 [(M-Glc)-H]^−^								+				
6	1.48	Apigenin-*C*-Hex-*O*-Hex-Hex^b^	265, 332	755 [M-H]^−^, 593 [(M-Hex)-H]^−^, 431 [(M-2 × Hex)-H]^−^, 341 [(M-2 × Hex-90)-H]^−^, 311 [(M-2 × Hex-120)-H]^−^, 313 [(M-2 × Hex-90-CO)-H]^−^, 283 [(M-2 × Hex-120-CO)-H]^−^						+						+
7	1.52	Apigenin-*C*-Hex-*O*-Hex-Hex^b^	265, 332	755 [M-H]^−^, 593 [(M-Hex)-H]^−^, 431 [(M-2 × Hex)-H]^−^, 341 [(M-2 × Hex-90)-H]^−^, 311 [(M-2 × Hex-120)-H]^−^, 313 [(M-2 × Hex-90-CO)-H]^−^, 283 [(M-2 × Hex-120-CO)-H]^−^		+				+		+				+
8	1.53	6-*O*-Feruloylglucose^a^	279, 307	355 [M-H]^−^, 193 [(M-Glc)-H]^−^	+											
9	1.54	Luteolin-6-*C*-Glc-7-*O*-Glc^a^	257, 328	609 [M-H]^−^, 447 [(M-Glc)-H]^−^, 357 [(M-90)-H]^−^, 327 [(M-120)-H]^−^, 329 [(M-90-CO)-H]^−^, 299 [(M-120-CO)-H]	+											
10	1.55	5-*O*-Caffeoylquinic acid^a^	326	353 [M-H]^−^, 191	+	+	+	+	+	+	+	+	+	+	+	+
11	1.63	Apigenin-*C*-Hex-*O*-Hex^b^	265, 332	593 [M-H]^−^, 431 [(M-Hex)-H]^−^, 341 [(M-Hex-90)-H]^−^, 311 [(M-Hex-120)-H]^−^, 313 [(M-Hex-90-CO)-H]^−^, 283 [(M-Hex-120-CO)-H]^−^	+	+	+	+		+		+		+		+
12	1.64	Eriodictyol-*O*-Hex^b^	289	449 [M-H]^−^, 287 [(M-Hex)-H]^−^							+					
13	1.64	Esculetin^a^	253, 298, 344	201 [M+Na]^+^, 179 [M+H]^+^								+	+			
14	1.65	1,3-Di-*O*-caffeoylquinic acid^a^	325	515 [M-H]^−^, 353, 191	+	+	+	+	+	+	+	+	+	+	+	+
15	1.66	Apigenin-*C*-Hex-*O*-Hex^b^	265, 332	593 [M-H]^−^, 431 [(M-Hex)-H]^−^, 341 [(M-Hex-90)-H]^−^, 311 [(M-Hex-120)-H]^−^, 313 [(M-Hex-90-CO)-H]^−^, 283 [(M-Hex-120-CO)-H]^−^	+					+	+					+
16	1.67	Eriodictyol-7-*O*-Glc (pyracanthoside)^a^	289	449 [M-H]^−^, 287 [(M-Glc)-H]^−^							+					
17	1.69	*cis*-Melilotoside^a^	291	325 [M-H]^−^, 163 [(M-Glc)-H]^−^											+	
18	1.75	Scopoletin-7-*O*-Glc (scopolin)^a^	328	377 [M+Na]^+^, 355 [M+H]^+^, 193 [(M-Glc)+H]^+^				+	+						+	
19	1.77	Apigenin-6-*C*-Glc-4″-*O*-Glc (isosaponarin)^a^	265, 333	593 [M-H]^−^, 431 [(M-Glc)-H]^−^, 341 [(M-Glc-90)-H]^−^, 311 [(M-Glc-120)-H]^−^, 313 [(M-Glc-90-CO)-H]^−^, 283 [(M-Glc-120-CO)-H]^−^		+	+	+						+		
20	1.78	Apigenin-*C*-Hex-*O*-Hex^b^	265, 332	593 [M-H]^−^, 431 [(M-Hex)-H]^−^, 341 [(M-Hex-90)-H]^−^, 311 [(M-Hex-120)-H]^−^, 313 [(M-Hex-90-CO)-H]^−^, 283 [(M-Hex-120-CO)-H]^−^	+		+	+								
21	1.81	Apigenin-6-*C*-Glc-7-*O*-Glc (saponarin)^a^	266, 333	593 [M-H]^−^, 431 [(M-Glc)-H]^−^, 341 [(M-Glc-90)-H]^−^, 311 [(M-Glc-120)-H]^−^, 313 [(M-Glc-90-CO)-H]^−^, 283 [(M-Glc-120-CO)-H]^−^		+	+							+	+	
22	1.82	5-*O*-Coumaroylquinic acid^a^	307	337 [M-H]^−^, 191				+	+						+	
23	1.82	Apigenin-*C*-Hex-*O*-HexA^b^	263, 330	607 [M-H]^−^, 431 [(M-HexA)-H]^−^, 341 [(M-HexA-90)-H]^−^, 311 [(M-HexA-120)-H]^−^, 313 [(M-HexA-90-CO)-H]^−^, 283 [(M-HexA-120-CO)-H]^−^			+	+								
24	1.83	Quercetin-3-*O*-^2^″RhaGlc (calendoflavobioside)^a^	253, 268, 351	609 [M-H]^−^, 463 [(M-Rha)-H]^−^, 301 [(M-Rha-Glc)-H]^−^		+	+			+		+	+			+
25	1.84	Quercetin-3-*O*-^4^″RhaGlc^a^	253, 268, 351	609 [M-H]^−^, 463 [(M-Rha)-H]^−^, 301 [(M-Rha-Glc)-H]^−^		+	+			+		+	+			+
26	1.85	Luteolin-6,8-di-*C*-Glc (lucenin-2)^a^	265, 340	609 [M-H]^−^, 519 [(M-90)-H]^−^, 489 [(M-Glc-120)-H]^−^, 429 [(M-Glc-2 × 90)-H]^−^, 399 [(M-Glc-120-90)-H]^−^, 369 [(M-Glc-2 × 120)-H]^−^, 401 [(M-Glc-2 × 90-CO)-H]^−^, 373 [(M-Glc-2 × 90-2 × CO)-H]^−^, 371 [(M-Glc-120-90-CO)-H]^−^, 343 [(M-Glc-120-90-2 × CO)-H]^−^, 341 [(M-Glc-2 × 120-CO)-H]^−^, 313 [(M-Glc-2 × 120-2 × CO)-H]^−^	+											
27	1.86	Apigenin-6-*C*-Ara-8-*C*-Glc (isoschaftoside)^a^	267, 333	563 [M-H]^−^, 503 [(M-60)-H]^−^, 473 [(M-90)-H]^−^, 443 [(M-120)-H]^−^, 413 [(M-90-60)-H]^−^, 383 [(M-120-60)-H]^−^, 353 [(M-120-90)-H]^−^, 325 [(M-120-90-CO)-H]^−^, 297 [(M-120-90-2 × CO)-H]^−^		+	+	+	+	+						
28	1.87	Apigenin-6,8-di-*C*-Glc (vicenin-2)^a^	269, 336	593 [M-H]^−^, 503 [(M-90)-H]^−^, 473 [(M-120)-H]^−^, 413 [(M-2 × 90)-H]^−^, 383 [(M-120-90)-H]^−^, 353 [(M-2 × 120)-H]^−^, 325 [(M-2 × 120-CO)-H]^−^, 297 [(M-2 × 120-2 × CO)-H]^−^										+	+	
29	1.89	6-Hydroxyluteolin-*O*-Hex-HexA^b^	271, 345	639 [M-H]^−^, 463 [(M-HexA)-H]^−^, 301 [(M-HexA-Hex)-H]^−^										+		
30	1.90	*trans*-Melilotoside^a^	275, 312	325 [M-H]^−^, 163 [(M-Glc)-H]^−^											+	
31	1.94	Apigenin-6-*C*-Glc-8-*C*-Ara (schaftoside)^a^	267, 333	563 [M-H]^−^, 503 [(M-60)-H]^−^, 473 [(M-90)-H]^−^, 443 [(M-120)-H]^−^, 413 [(M-90-60)-H]^−^, 383 [(M-120-60)-H]^−^, 353 [(M-120-90)-H]^−^, 325 [(M-120-90-CO)-H]^−^, 297 [(M-120-90-2 × CO)-H]^−^			+	+	+		+					
32	1.95	Quercetin-*O*-dHex-*O*-Hex^b^	252, 267, 352	609 [M-H]^−^, 463 [(M-dHex)-H]^−^, 301 [(M-dHex-Hex)-H]^−^						+			+			+
33	1.96	Coumarin-*O*-Hex^b^	324	509 [M+Na]^+^, 487 [M+H]^+^, 325 [(M-Hex)+H]^+^											+	
34	1.96	Chrysoeriol-6,8-di-*C*-Glc (stellarin-2)^a^		623 [M-H]^−^, 533 [(M-90)-H]^−^, 503 [(M-Glc-120)-H]^−^, 443 [(M-Glc-2 × 90)-H]^−^, 413 [(M-Glc-120-90)-H]^−^, 383 [(M-Glc-2 × 120)-H]^−^, 415 [(M-Glc-2 × 90-CO)-H]^−^, 387 [(M-Glc-2 × 90-2 × CO)-H]^−^, 385 [(M-Glc-120-90-CO)-H]^−^, 357 [(M-Glc-120-90-2 × CO)-H]^−^, 355 [(M-Glc-2 × 120-CO)-H]^−^, 327 [(M-Glc-2 × 120-2 × CO)-H]^−^	+											
35	1.99	Luteolin-6-*C*-Glc-8-*C*-Xyl (lucenin-3)^a^	266, 340	579 [M-H]^−^, 519 [(M-60)-H]^−^, 489 [(M-90)-H]^−^, 459 [(M-120)-H]^−^, 429 [(M-90-60)-H]^−^, 399 [(M-120-60)-H]^−^, 369 [(M-120-90)-H]^−^, 401 [(M-90-60-CO)-H]^−^, 373 [(M-90-60-2 × CO)-H]^−^, 371 [(M-120-60-CO)-H]^−^, 343 [(M-90-60-2 × CO)-H]^−^, 341 [(M-120-90-CO)-H]^−^, 313 [(M-120-90-2 × CO)-H]^−^										+		
36	2.00	Luteolin-6-*C*-Xyl-8-*C*-Glc (lucenin-1)^a^	265, 341	579 [M-H]^−^, 519 [(M-60)-H]^−^, 489 [(M-90)-H]^−^, 459 [(M-120)-H]^−^, 429 [(M-90-60)-H]^−^, 399 [(M-120-60)-H]^−^, 369 [(M-120-90)-H]^−^, 401 [(M-90-60-CO)-H]^−^, 373 [(M-90-60-2 × CO)-H]^−^, 371 [(M-120-60-CO)-H]^−^, 343 [(M-90-60-2 × CO)-H]^−^, 341 [(M-120-90-CO)-H]^−^, 313 [(M-120-90-2 × CO)-H]^−^										+		
37	2.01	Luteolin-di-*C*-Hex^b^	265, 340	609 [M-H]^−^, 519 [(M-90)-H]^−^, 489 [(M-120)-H]^−^, 429 [(M-2 × 90)-H]^−^, 399 [(M-120-90)-H]^−^, 369 [(M-2 × 120)-H]^−^, 401 [(M-2 × 90-CO)-H]^−^, 373 [(M-2 × 90-2 × CO)-H]^−^, 371 [(M-120-90-CO)-H]^−^, 343 [(M-120-90-2 × CO)-H]^−^, 341 [(M-2 × 120-CO)-H]^−^, 313 [(M-2 × 120-2 × CO)-H]^−^										+		
38	2.02	Quercetin-3-*O*-^6^″RhaGlc (rutin)^a^	253, 268, 351	609 [M-H]^−^, 463 [(M-Rha)-H]^−^, 301 [(M-Rha-Glc)-H]^−^	+	+	+	+	+	+	+	+	+		+	+
39	2.04	6-Hydroxyluteolin-*O*-HexA^b^	270, 344	477 [M-H]^−^, 301 [(M-HexA)-H]^−^										+		
40	2.05	Quercetin-3-*O*-Glc (isoquercitrin)^a^	256, 268, 351	463 [M-H]^−^, 301 [(M-Gal)-H]^−^	+	+	+	+	+	+	+	+	+		+	+
41	2.06	Luteolin-6-*C*-Glc (isoorientin)^a^	266, 342	447 [M-H]^−^, 357 [(M-90)-H]^−^, 327 [(M-120)-H]^−^, 329 [(M-90-CO)-H]^−^, 299 [(M-120-CO)-H]	+									+		
42	2.08	Quercetin-3-*O*-Gal (hyperoside)^a^	255, 267, 352	463 [M-H]^−^, 301 [(M-Glc)-H]^−^		+	+				+	+	+		+	+
43	2.11	Luteolin-7-*O*-GlcA^a^	266, 346	461 [M-H]^−^, 285 [(M-GlcA)-H]^−^										+		
44	2.14	3,4-Di-*O*-caffeoylquinic acid^a^	325	515 [M-H]^−^, 353, 191	+	+	+	+	+	+	+	+	+	+	+	+
45	2.15	Kaempferol-3-*O*-^6^″RhaGlc (nicotiflorin)^a^	266, 341	593 [M-H]^−^, 447 [(M-Rha)-H]^−^, 285 [(M-Rha-Glc)-H]^−^	+	+		+	+	+		+	+			+
46	2.16	Isorhamnetin-3-*O*-^6^″RhaGlc (narcissin)^a^	254, 354	623 [M-H]^−^, 477 [(M-Rha)-H]^−^, 315 [(M-Rha-Glc)-H]^−^	+	+	+	+	+	+	+		+		+	+
47	2.17	Luteolin-7-*O*-Glc (cynaroside)^a^	266, 340	447 [M-H]^−^, 285 [(M-Glc)-H]^−^										+	+	
48	2.17	Quercetagetin-dimethyl ester-*O*-dHex-Hex^Bb^	257, 268, 353	653 [M-H]^−^, 507 [(M-dHex)-H]^−^, 345 [(M-dHex-Hex)-H]^−^, 330, 315			+									
49	2.18	Apigenin-6-*C*-Glc (isovitexin)^a^	267, 344	431 [M-H]^−^, 341 [(M-90)-H]^−^, 311 [(M-120)-H]^−^, 313 [(M-90-CO)-H]^−^, 283 [(M-120-CO)-H]^−^										+		
50	2.22	3,5-Di-*O*-caffeoylquinic acid^a^	325	515 [M-H]^−^, 353, 191	+	+	+	+	+	+	+	+	+	+	+	+
51	2.25	Luteolin-*O*-Hex^b^	265, 340	447 [M-H]^−^, 285 [(M-Hex)-H]^−^										+	+	
52	2.25	1,5-Di-*O*-caffeoylquinic acid^a^	325	515 [M-H]^−^, 353, 191											+	
53	2.26	Kaempferol-3-*O*-Glc (astragalin)^a^	265, 340	447 [M-H]^−^, 285 [(M-Glc)-H]^−^	+	+		+		+	+	+	+			+
54	2.27	Isorhamnetin-3-*O*-Glc^a^	255, 353	477 [M-H]^−^, 315 [(M-Glc)-H]^−^	+	+	+	+		+	+		+		+	+
55	2.28	Apigenin-7-*O*-Glc (cosmosiin)^a^	268, 337	431 [M-H]^−^, 269 [(M-Glc)-H]^−^						+		+		+		+
56	2.29	Luteolin-*O*-Hex^b^	265, 340	447 [M-H]^−^, 285 [(M-Hex)-H]^−^										+	+	
57	2.30	Kaempferol-methyl ester-*O*-Hex^b^	270, 337	461 [M-H]^−^, 299 [(M-Hex)-H]^−^, 284			+									
58	2.31	4,5-Di-*O*-caffeoylquinic acid^a^	325	515 [M-H]^−^, 353, 191	+	+	+	+	+	+	+	+	+	+	+	+
59	2.32	Luteolin-trimethyl ester-*O*-Hex^b^	269, 346	505 [M-H]^−^, 343 [(M-Hex)-H]^−^										+		
60	2.32	Quercetagetin-dimethyl ester-*O*-Hex^b^	254, 269, 354	507 [M-H]^−^, 345 [(M-Hex)-H]^−^, 330, 315			+									
61	2.34	Chrysoeriol-7-*O*-Glc^a^	283, 327	461 [M-H]^−^, 299 [(M-Glc)-H]^−^	+											+
62	2.35	Chrysoeriol-7-*O*-GlcA^a^	269, 345	475 [M-H]^−^, 299 [(M-GlcA)-H]^−^										+		
63	2.36	Scutellarein-7-*O*-Glc^a^	268, 325	447 [M-H]^−^, 285 [(M-Glc)-H]^−^					+							
64	2.37	Isorhamnetin-*O*-Hex^b^	255, 354	477 [M-H]^−^, 315 [(M-Hex)-H]^−^											+	
65	2.37	Quercetin-dimethyl ester-*O*-Hex^b^	255, 270, 353	491 [M-H]^−^, 329 [(M-Hex)-H]^−^, 314, 299			+									
66	2.38	Kaempferol-methyl ester-*O*-Hex^b^	270, 337	461 [M-H]^−^, 299 [(M-Hex)-H]^−^, 284				+								
67	2.38	Chrysoeriol-*O*-Hex^b^	283, 327	461 [M-H]^−^, 299 [(M-Hex)-H]^−^	+											
68	2.38	5,7,3′-Trihydroxy-6,4′-dimetoxyflavone-7-*O*-GlcA^b^	251, 267, 346	505 [M-H]^−^, 329 [(M-GlcA)-H]^−^										+		
69	2.39	Hispidulin-7-*O*-Glc (homoplantaginin)^a^	275, 336	461 [M-H]^−^, 299 [(M-Glc)-H]^−^					+							
70	2.39	Apigenin-methyl ester-*O*-Hex^b^	266, 334	461 [M-H]^−^, 299 [(M-Hex)-H]^−^, 284										+		
71	2.45	Isorhamnetin-3-*O*-dHex^a^	255, 353	447 [M-H]^−^, 315 [(M-dHex)-H]^−^											+	
72	2.46	Nepetin-7-*O*-Glc (nepetrin)^a^	254, 273, 342	477 [M-H]^−^, 315 [(M-Glc)-H]^−^								+				
73	2.56	Kaempferol-methyl ester-*O*-Hex^b^	268, 338	461 [M-H]^−^, 299 [(M-Hex)-H]^−^, 284		+										
74	2.59	Lacarol^b^	258, 322	339 [M+HCOO]^+^, 317 [M+Na]^+^, 295 [M+H]^+^, 277 [(M-H_2_O)+H]^+^, 209 [(M-C_5_H_10_O)+H]^+^, 195 [(M-C_5_H_10_O-CH_2_)+H]^+^					+						+	
75	2.65	Sakuranetin^a^	287	285 [M-H]^−^									+			
76	2.67	Scutellarein^a^	286, 310	257 [M-H]^−^									+			
77	2.70	Luteolin-methyl ester-*O*-Hex^b^	268, 327	461 [M-H]^−^, 299 [(M-Hex)-H]^−^, 284	+											
78	2.72	Quercetin^a^	255, 267, 353	301 [M-H]^−^		+				+	+		+			
79	2.77	6-Hydroxyluteolin^a^	272, 346	301 [M-H]^−^								+		+		
80	2.78	Eriodictyol^a^	289	287 [M-H]^−^								+				
81	2.85	Luteolin^a^	266, 345	285 [M-H]^−^	+							+		+		+
82	2.88	Nepetin^a^	255, 272, 343	315 [M-H]^−^								+				
83	2.98	Desoxylacarol^b^	258, 319	323 [M+HCOO]^+^, 301 [M+Na]^+^, 279 [M+H]^+^, 261 [(M-H_2_O)+H]^+^, 193 [(M-C_5_H_10_O)+H]^+^, 179 [(M-C_5_H_10_O-CH_2_)+H]^+^					+						+	
84	3.02	6,8-Dihydroxyluteolin-dimethyl ester	275, 341	345 [M-H]^−^, 330, 315								+				
85	3.21	Davidigenin^a^	275, 312, 364	257 [M-H]^−^									+			
86	3.24	Apigenin^a^	269, 337	269 [M-H]^−^				+		+		+	+	+		+
87	3.25	Chrysoeriol^a^	267, 344	299 [M-H]^−^								+		+		+
88	3.26	Kaempferol^a^		285 [M-H]^−^	+											
89	3.26	Hispidulin^a^	275, 336	299 [M-H]^−^						+		+				+
90	3.27	Isorhamnetin^a^		315 [M-H]^−^		+										
91	3.30	Methyllacarol^b^	262, 324	353 [M+HCOO]^+^, 331 [M+Na]^+^, 309 [M+H]^+^, 291 [(M-H_2_O)+H]^+^, 223 [(M-C_5_H_10_O)+H]^+^, 209 [(M-C_5_H_10_O-CH_2_)+H]^+^, 195 [(M-C_5_H_10_O-2 × CH_2_)+H]^+^					+						+	
92	3.32	5,7,3′-Trihydroxy-6,4′-dimetoxyflavone^b^	272, 343	329 [M-H]^−^, 314, 299										+		
93	3.35	Chrysin^a^	278, 310	253 [M-H]^−^									+			
94	3.38	Jaceosidin^a^	250, 272, 344	329 [M-H]^−^					+	+		+				+
95	3.75	Eupatilin^a^	245, 274, 341	343 [M-H]^−^				+	+	+						+
96	3.78	5,4′-Dihydroxy-6,7,3′-trimetoxyflavone^b^	273, 341	343 [M-H]^−^, 328, 313, 298										+		
97	3.80	Cirsiliol^a^	275, 335	329 [M-H]^−^								+				
98	3.85	Davidigenin-methyl ester^b^	275, 311	271 [M-H]^−^, 256									+			
99	3.87	Eupatorin^a^	273, 343	343 [M-H]^−^								+				+
100	3.93	Quercetagetin-tetramethyl ester^b^	272, 354	373 [M-H]^−^, 358, 343, 328, 313			+						+			
101	3.97	6-Hydroxyluteolin-dimethyl ester^b^	275, 340	329 [M-H]^−^, 314, 299								+				
102	3.98	Chrysosplenetin^a^	255, 350	373 [M-H]^−^					+	+						+
103	4.01	3′-Hydroxygenkwanin^a^	272, 331	299 [M-H]^−^								+				
104	4.06	Genkwanin^a^	270, 335	283 [M-H]^−^				+	+	+		+				+
105	4.10	Davidigenin-dimethyl ester^b^	270, 310	285 [M-H]^−^, 270, 255									+			
106	4.12	5,3′-Hydroxy-7,4′-dimetoxyflavone^a^	275, 331	313 [M-H]^−^										+		
107	4.14	Velutin^a^	252, 269, 341	313 [M-H]^−^								+				
108	4.18	6-Hydroxyluteolin-trimethyl ester^b^	274, 343	357 [M-H]^−^, 342, 327, 312									+			
109	4.23	5-Desmethylnobiletin^a^	271, 341	387 [M-H]^−^								+				
110	4.25	5-Desmethylsinensetin^a^	274, 343	357 [M-H]^−^					+					+		+
111	4.39	Sinensetin^a^	240, 330	371 [M-H]^−^												+
112	4.90	Davidigenin-trimethyl ester^b^	268, 308	299 [M-H]^−^, 284, 269, 254									+			

a *compound identification was based on comparison to standard*.

b *compound identification was based on interpretation of UV and MS spectral data and comparison with literature data. “+,” presence of compound; Ara, arabinose; dHex, desoxyhexose; Glc, glucose; GlcA, glucuronic acid; Hex, hexose; HexA, hexuronic acid; Rha, rhamnose;^2^″RhaGlc, 2″-rhamnosyl-glycose (neohesperidose);^4^″RhaGlc, 4″-rhamnosyl-glycose;^6^″RhaGlc, 6″-rhamnosyl-glycose (rutinose); Xyl, xylose*.

Fourteen phenylpropanoids were found in *Artemisia* extracts in the present study. Compounds **1** and **10** were confirmed as mono-*O*-caffeoylquinic acids (CQA), according to their UV-spectra and [M–H]^−^ ion with *m*/*z* 353, and differentiated as 4-*O*-CQA and 5-*O*-CQA, respectively, after comparing with standards. Similar UV-patterns were found for **14**, **44**, **50**, **52**, and **58** resulting in [M–H]^−^ ion with *m*/*z* 515 and daughter ions with *m*/*z* 353 and 191.

Chromatographic behavior was identical to known di-*O*-caffeoylquinic acids as 1,3-di-*O*-CQA, 3,4-di-*O*-CQA, 3,5-di-*O*-CQA, 1,5-di-*O*-CQA and 4,5-di-*O*-CQA, respectively. Compound **22** was characterized by the different UV-maxima (307 nm) and [M–H]^−^ ion with *m*/*z* 337 specific for the standard of 5-*O*-coumaroylquinic acid. Compounds **1**, **10**, **14**, **44**, **50**, and **58** were found in all *Artemisia* extracts, in contrast to **22**, detected in *A. latifolia* and *A. tanacetifolia*, and **52**, observed in *A. tanacetifolia* only. Phenylpropanoid glycosides were presented by 6-*O*-feruloylglucose (**8**) in *A. anethifolia*, two isomers of melilotoside (**17**, **30**) in *A. tanacetifolia*, dihydroferulic acid-*O*-glycoside (**3**) in *A. anethifolia* and caffeic acid-*O*-glycoside (**5**) in *A. messerschmidtiana*. Compounds **8**, **17**, and **30** were identified by comparing their chromatographic and spectral data with standards. **3** and **5** were putatively identified using literature data (Vallverdú-Queralt et al., 2010).

The only simple phenolic acid *O*-glycoside from *A. sericea* had a UV-maxima at 255, 293 nm and MS-ions (*m*/*z* 315 → 153), and was identified as protocatechuic acid-*O*-glycoside (Chen et al., 2012).

A total of 7 coumarins, of simple structure and hemiterpene-coupled substances, were identified using MS detection with positive ionization mode. Component **14**, discovered in five *Artemisia* extracts, had a pseudomolecular ion with *m*/*z* 325 [M+H]^+^, and an adduct ion with *m*/*z* 347 [M+Na]^+^ as well as a deglucosidated fragment with *m*/*z* 163. Similarity to the standard UV-pattern and t_R_ value, allowed us to identify **14** as umbelliferone-7-*O*-glucoside or skimmin. The values of *m*/*z* for the pseudomolecular and adduct ions and deglucosidated fragment of **18**, were 30 Da more than **14**, indicating a close similarity to **14** structures, with additional methoxy-function. After comparing with the reference compound, it was summarized as scopoletin-7-*O*-glucoside or scopolin, and was detected in *A. anethifolia, A. integrifolia, A. latifolia* and *A. tanacetifolia*. Chromatographic behavior and spectral data of **13**, from *A. messerschmidtiana*, was identical to esculetin.

Compounds **74**, **83**, and **91**, found in *A. latifolia* and *A. tanacetifolia*, were putatively identified as rare coumarin-hemiterpene ethers. Specific MS/MS patterns of **74** showed an [M+H]^+^ ion with *m*/*z* 295, two adduct ions with *m*/*z* 317 [M+Na]^+^ and 339 [M+HCOO]^+^ and fragments initiate by the elimination of water {*m*/*z* 277 [(M–H_2_O)+H]^+^} and 4′-hydroxy-3′-methylbythoxy-group {*m*/*z* 209 [(M–C_5_H_10_O)+H]^+^; 195 [(M–C_5_H_10_O–CH_2_)+H]^+^}. Thus, it was characterized as 5-hydroxy-7-methoxy-8-(4′-hydroxy-3′-methylbythoxy)coumarin or lacarol, previously isolated from *A. laciniata* (Hofer et al., 1986) and *A. armeniaca* (Mojarrab et al., 2011). Compound **83** is similar to **74** MS-fragmentation, with an *m*/*z* difference of 16 Da less for all general fragments, indicating a compound structure as the desoxy-analog of lacarol or 7-methoxy-8-(4′-hydroxy-3′-methylbythoxy)coumarin (desoxylacarol). The nature of compound **91** was determined to be methyllacarol, or 5,7-dimethoxy-8-(4′-hydroxy-3′-methylbythoxy)coumarin, due to the difference of *m*/*z* values of 14 Da more for the principal MS-fragments, pointing to additional methyl-function (Hofer et al., 1986).

The extract of *A. palustris* contained four components (**85**, **98**, **105**, **112**), with a particular UV pattern, distinctive for dihydrochalcones, described early for *A. dracunculus* (Logendra et al., 2006). Compound **85**, which gave a pseudomolecular ion [M–H]^−^ with *m*/*z* 257 in the negative ionization mode, and adsorption bands in UV spectrum at 275, 312, and 364 nm, was identified as 4,2′,4′-trihydroxydihydrochalcone, or davidigenin, after comparison with standard (Jensen et al., 1977; Logendra et al., 2006). Three related components (**98**, **105**, **112**), with close spectral data, were clearly identified as *O*-methyl esters of davidigenin. Their MS fragments included [M–H]^−^ ions (*m*/*z* 271, 285, 299) and fragments caused by sequential elimination of methyl-functions, like *m*/*z* 271 → 256 for **98**; *m*/*z* 285 → 270 → 255 for **105**; and *m*/*z* 299 → 284 → 269 → 254 for **112**. Thus, they were identified as mono- (**98**), di- (**105**) and tri-*O*-methyl ester of davidigenin (**112**).

Flavone derivatives of *Artemisia* extracts were the most representative group of phenolics, which totaled 60 compounds, including 36 glycosides and 24 aglycones. Glycosidic components were derivatives of apigenin (14 compounds), luteolin (12), chrysoeriol (4), 6-hydroxyluteolin (2) and some minor flavones as *O*-, *C*- and *C*,*O*-glucosides with arabinose, glucose, glucuronic acid and xylose residues.

Apigenin glycosides were detected in all species, except *A. palustris*, and unambiguous identification was done after comparing spectral data with standards for **19** as apigenin-6-*C*-glucoside-4″-*O*-glucoside (isosaponarin), **21** as apigenin-6-*C*-glucoside-7-*O*-glucoside (saponarin), **27** as apigenin-6-*C*-arabinoside-8-*C*-glucoside (isoschaftoside), **28** as apigenin-6,8-di-*C*-glucoside (vicenin-2), **31** as apigenin-6-*C*-glucoside-8-*C*-arabinoside (schaftoside), **49** as apigenin-6-*C*-glucoside (isovitexin), and **55** as apigenin-7-*O*-glucoside (cosmosiin). Two compounds, **6** and **7**, with similar UV- and MS-parameters, gave a pseudomolecular ion [M–H]^−^ with *m*/*z* 755 and two ions initiated by elimination of two *O*-bonded hexose moieties (*m*/*z* 755 → 593 → 431). Detection of an ion with *m*/*z* 341 [(M−2 × Hex−90)–H]^−^, 311 [(M−2 × Hex−120)–H]^−^, 313 [(M−2 × Hex−90–CO)–H]^−^, and 283 [(M−2 × Hex−120–CO)–H]^−^ was characteristic for *C*-glycosides (Han et al., 2008). Thus, both compounds were isomeric and their structures were determined to be apigenin-*C*-hexoside-*O*-hexosyl-hexoside (or apigenin-*C*-hexoside-di-*O*-hexoside). Compounds **11**, **15**, and **20** gave [M–H]^−^ ion with *m*/*z* 593 and a series of ions with *m*/*z* 431 [(M–Hex)–H]^−^, 341 [(M-Hex−90)–H]^−^, 311 [(M–Hex−120)–H]^−^, 313 [(M–Hex−90–CO)–H]^−^, and 283 [(M–Hex−120–CO)–H]^−^, suggesting their structures were apigenin-*C*-hexoside-*O*-hexoside. Compound **23**, detected in *A. desertorum* and *A. integrifolia*, yielded an ion [M–H]^−^ with *m*/*z* 607, and the ion of the de-hexuronidated fragment was *m*/*z* 431 [(M–HexA)–H]^−^, as well as *C*-hexose-specific ions with *m*/*z* 341 [(M–HexA−90)–H]^−^, 311 [(M–HexA−120)–H]^−^, 313 [(M–HexA−90–CO)–H]^−^, and 283 [(M–HexA−120–CO)–H]^−^, characterized the structure as apigenin-*C*-hexoside-*O*-hexuronide. Lipophilic *O*-hexoside **70** from *A. tanacetifolia*, with a UV spectral maxima at 266 and 334 nm, and [M–H]^−^ ion with *m*/*z* 461 and de-hexosidated fragment with *m*/*z* 299 was interpreted as apigenin monomethyl ester-*O*-hexoside.

Twelve luteolin derivatives were discovered only in five *Artemisia* species, with maximal occurrence in *A. sericea* (7 compounds). Among them, seven glycosides were identified, with standards, as luteolin-6-*C*-glucoside-7-*O*-glucoside (**9**), luteolin-6,8-di-*C*-glucoside or lucenin-2 (**26**), luteolin-6-*C*-glucoside-8-*C*-xyloside or lucenin-3 (**35**), luteolin-6-*C*-xyloside-8-*C*-glucoside or lucenin-1 (**36**), luteolin-6-*C*-glucoside or isoorientin (**41**), luteolin-7-*O*-glucuronide (**43**) and luteolin-7-*O*-glucoside or cymaroside (**47**). Compound **37** gave an [M–H]^−^ ion with *m*/*z* 609, and the fragments belonged to flavone-di-*C*-hexosides like 519 [(M−90)–H]^−^, 489 [(M−120)–H]^−^, 429 [(M−2 × 90)–H]^−^, 399 [(M−120–90)–H]^−^, 369 [(M−2 × 120)–H]^−^, 401 [(M−2 × 90–CO)–H]^−^, 373 [(M−2 × 90–2 × CO)–H]^−^, 371 [(M−120–90–CO)–H]^−^, 343 [(M−120–90-2 × CO)–H]^−^, 341 [(M−2 × 120–CO)–H]^−^, and 313 [(M−2 × 120–2 × CO)–H]^−^ (Han et al., 2008). The most likely structure of **37** was luteolin-di-*C*-hexoside, or lucenin-2, that was previously detected in *A. monosperma* and *A. herba-alba* (Saleh et al., 1985). Two luteolin-*O*-hexosides, **51** and **56** (*m*/*z* 447 → 285), were discovered in *A. sericea* and *A. tanacetifolia*. Luteolin-trimethyl ester-*O*-hexoside (**59**; *m*/*z* 505 → 343) and luteolin-methyl ester-*O*-hexoside (**77**; *m*/*z* 461 → 299) were the components of *A. sericea* and *A. anethifolia*, respectively.

Three chrysoeriol glycosides, from *A. anethifolia, A. sericea* and *A. umbrosa*, were determined to be chrysoeriol-6,8-di-*C*-glycoside (**34**), chrysoeriol-7-*O*-glucoside (**61**) and chrysoeriol-7-*O*-glucuronide (**62**) after comparison with standards. Compound **67**, with UV maxima at 283 and 327 nm and [M–H]^−^ ion with *m*/*z* 461 and fragment with *m*/*z* 299 [(M–Hex)–H]^−^, was identified as chrysoeriol-*O*-hexoside. Only two 6-hydroxyluteolin glycosides (**29**, **39**) were detected and both were in *A. sericea*. Compound **29** gave an [M–H]^−^ ion with *m*/*z* 639 and two principal fragments caused by elimination of the residues of hexuronic acid (*m*/*z* 463) and hexose (*m*/*z* 301), demonstrating its possible structure as 6-hydroxyluteolin-*O*-hexoside-*O*-hexuronide (or 6-hydroxyluteolin-*O*-hexuronyl-hexoside) (Es-Safi et al., 2005). Compound **39** exhibited an [M–H]^−^ ion with *m*/*z* 477 and a de-hexuronidated fragment with *m*/*z* 301 and was identified as 6-hydroxyluteolin-*O*-hexuronide. Scutellarein-7-*O*-glucoside (**63**) and hispidulin-7-*O*-glucoside, or homoplantaginin (**69**), from *A. latifolia* and nepetin-7-*O*-glucoside or nepetrin (**72**) from *A. messerschmidtiana* were identified with standards. Compound **68** from *A. sericea*, with UV maxima at 251, 267 and 346 nm and MS fragments with *m*/*z* 505 [M-H]^−^ and 329 [(M–GlcA)–H]^−^, was determined as 5,7,3′-trihydroxy-6,4′-dimetoxyflavone-7-*O*-glucuronide that has already been found in that species (Wang Q. et al., 2016).

The aglycones with a flavone structure, the largest group of flavonoids, included 24 compounds detected in 8 *Artemisia* species, except *A. commutata, A. desertorum, A. macrocephala* and *A. tanacetifolia*. The vast majority of them have been identified comparing chromatographic and spectral data with standards, identifying scutellarein (**76**), 6-hydroxyluteolin (**79**), luteolin (**81**), nepetin (**82**), apigenin (**86**), chrysoeriol (**87**), hispidulin (**89**), chrysin (**93**), jaceosidin (**94**), eupatilin (**95**), cirsiliol (**97**), eupatorin (**99**), 3′-hydroxygenkwanin (**103**), genkwanin (**104**), 5,3′-hydroxy-7,4′-dimetoxyflavone (**106**), velutin (**107**), 5-desmethylnobiletin (**109**), 5-desmethylsinensetin (**110**) and sinensetin (**111**). Compound **84** from *A. messerschmidtiana* gave an [M–H]^−^ ion with *m*/*z* 345, and fragments with *m*/*z* 330 and 315 indicating the presence of two methoxy-groups and was tentatively identified as 6,8-dihydroxyluteolin-dimethyl ester. Compound **92**, from *A. sericea* with UV maxima at 272 and 343 nm, gave as MS fragment with *m*/*z* 329 [M–H]^−^ and two demethylated ions with *m*/*z* 314 and 299, indicating the existence of two CH_3_ groups. It was identified with 5,7,3′-trihydroxy-6,4′-dimetoxyflavone, which was reported as *A. frigida* previously (Wang Q. et al., 2016). Flavone aglycone **96** gave an [M–H]^−^ ion with *m*/*z* 343 and fragments with *m*/*z* 328, 313 and 298, caused by elimination of three methyl-groups, and was determined to be 5,4′-dihydroxy-6,7,3′-trimetoxyflavone, also found in *A. frigida* (Wang Q. et al., 2016). Compounds **101** from *A. messerschmidtiana* and **108** from *A. palustris* were provisionally identified as 6-hydroxyluteolin-dimethyl ester and 6-hydroxyluteolin-trimethyl ester, due to their UV-patterns and MS fragmentation (*m*/*z* 329 → 314 → 299 for **101**; *m*/*z* 357 → 342 → 327 → 312 for **108**).

Flavonol glycosides were derivatives of kaempferol (5 compounds), quercetin (7), isorhamnetin (4) and quercetagetin (2), with only an *O*-type of glycosidic bond. Kaempferol glycosides were identified using the standards of nicotiflorin (kaempferol-3-*O*-rutinoside; **45**) and astragalin (kaempferol-3-*O*-glycoside; **53**). Three components (**57**, **66**, **73**), with close MS fragmentation (*m*/*z* 461, 299) and UV spectra, were assigned the structure of kaempferol-methyl ester-*O*-hexoside isomers.

The majority of quercetin glycosides were adequately identified using reference compounds with quercetin-3-*O*-neohesperidoside or calendoflavobioside (**24**), quercetin-3-*O*-(4″-rhamnosyl)glycoside (**25**), quercetin-3-*O*-rutinoside or rutin (**38**), quercetin-3-*O*-glucoside or isoquercitrin (**40**) and quercetin-3-*O*-galactoside or hyperoside (**42**). Compound **32**, detected in *A. leucophylla, A. palustris* and *A. umbrosa*, gave an [M–H]^−^ ion with *m*/*z* 609, and two fragments, induced by loss of desoxyhexose (*m*/*z* 463) and hexose moieties (*m*/*z* 301). The structure of **32** was assigned as quercetin-*O*-desoxyhexoside-*O*-hexoside. Compound **65** was found only in *A. desertorum* and produced an [M–H]^−^ ion with *m*/*z* 491, dehexosylated fragment [(M–Hex)–H]^−^ with *m*/*z* 329 and demethylated ions with *m*/*z* 314 and 299, demonstrating its structure as quercetin-dimethyl ester-*O*-hexoside.

Isorhamnetin-3-*O*-rutinoside or narcissin (**46**), isorhamnetin-3-*O*-glucoside (**54**) and isorhamnetin-3-*O*-galactoside (**71**) were found by comparing their properties with standards. Compound **64** was identified on the basis of UV spectrum (255, 354 nm) and MS pattern (*m*/*z* 477 → 315) as isorhamnetin-*O*-hexoside.

Two components of *A. desertorum* (**48**, **60**) have similar UV spectra but different MS patterns. Compound **48** gave a deprotonated ion with *m*/*z* 653 and two fragments with *m*/*z* 507 and 345, caused by elimination of desoxyhexose and hexose, but **60** has two principal fragments with *m*/*z* 507 [M–H]^−^ and 345 [(M–Hex)–H]^−^. The presence of the ions with *m*/*z* 330 and 315 indicated the loss of two methyl-groups in an aglycone moiety, assigned as quercetagetin-dimethyl ester. Thus, the predicted structures of the compounds were quercetagetin-dimethyl ester-*O*-desoxyhexosyl-hexoside for **48** and quercetagetin-dimethyl ester-*O*-hexoside for **60**.

Only five flavonol aglycones were detected in seven *Artemisia* species, among them were quercetin (**78**), kaempferol (**88**), isorhamnetin (**90**) and chrysosplenetin (**102**), easily identified with standards. Compound **100**, from *A. desertorum* and *A. Palustris*, gave an [M–H]^−^ ion with *m*/*z* 373 and four fragments, after sequential loss of methyl-groups, with *m*/*z* 358, 343, 328, and 313. The component was classified as a tetramethoxy-substituted quercetagetin derivative (Valant-Vetschera and Wollenweber, 1995; Valant-Vetschera et al., 2003).

Two flavanone glycosides were found in *A. Macrocephala*, identified as pyracanthoside (eriodictyol-7-*O*-glycoside; **16**) by comparing with standard, and eriodictyol-*O*-hexoside (**12**), based on its spectral data. Aglycones of flavanone were sakuranetin (**75**) in *A. palustris* and eriodictyol (**80**) in *A. messerschmidtiana*.

### Quantitative analysis of 15 phenolic compounds by microcolumn HPLC-DAD

**Figure 5:**
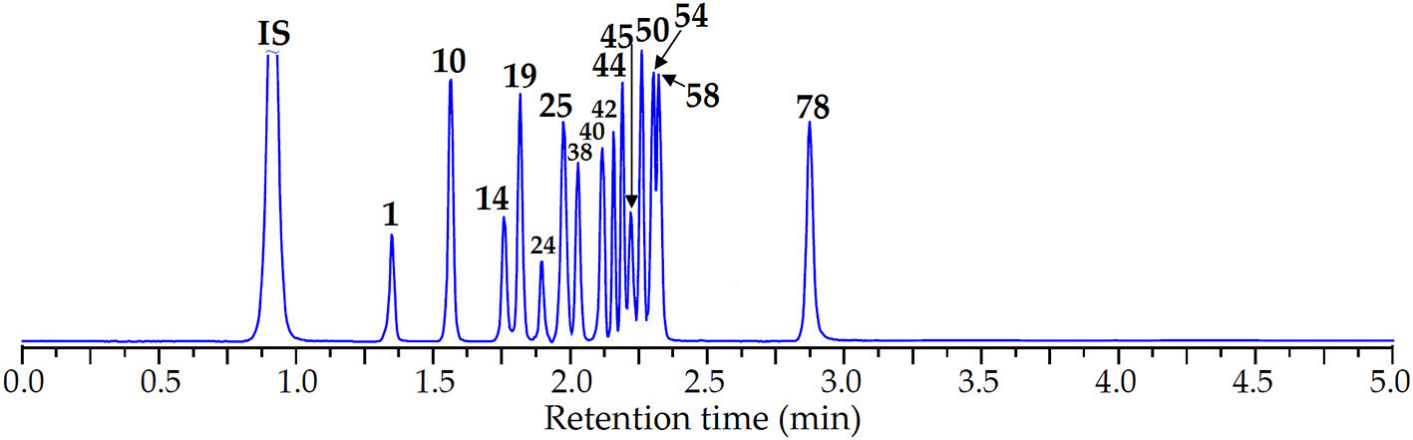
Microcolumn RP-HPLC-DAD chromatogram of the reference mixture of 15 compounds at 280 nm. Compounds are numbered as listed in Table 2. IS, internal standard (scopoletin-7-*O*-neohesperidoside).

We identified 15 phenolic compounds, including six caffeoylquinic acids (**1**, **10**, **14**, **44**, **50**, **58**) and nine flavonoids (**19**, **24**, **25**, **38**, **40**, **42**, **45**, **54**, **78**) that were chosen for their simultaneous quantitative estimation in *Artemisia* extracts by microcolumn HPLC-DAD assay. Total analysis time was 5 min and scopoletin-7-*O*-neohesperidoside was used as the internal standard to enhance the quality of method validation criteria. Chromatogram samples of the reference substances mixture and *Artemisia* extracts are illustrated in Figures 5, 3, respectively. Analyses of calibration curve validation data of 15 compounds, demonstrated their satisfactory linearity with correlation coefficient (*r*^2^) values 0.9998–0.9999 and standard deviation (*S*_YX_) values 1.14·10^−3^−9.26·10^−3^ (Table 3).

**Table 3:** Regression equations, correlation coefficients (*r*^2^), standard deviation (*S*_YX_), limits of detection (LOD), limits of quantitative (LOQ), and linear ranges for 15 compounds.

**Compound**	**Regression equation**	***r*^2^**	***S*_YX_**	**LOQ (μg/mL)**	**LOD (μg/mL)**	**Linear range (μg/mL)**
4-*O*-CQA (**1**)	*y* = 0.046·*x* – 0.045	0.9999	9.26·10^−3^	0.68	2.06	2.5–500.0
5-*O*-CQA (**10**)	*y* = 0.058·*x* – 0.043	0.9999	1.53·10^−3^	0.12	0.36	1.0–500.0
1,3-Di-*O*-CQA (**14**)	*y* = 0.046·*x* – 0.045	0.9999	6.99·10^−3^	0.51	1.55	2.5–500.0
A-6-*C*-Glc-4″-*O*-Glc (**19**)	*y* = 0.029·*x* + 0.050	0.9999	7.37·10^−3^	0.14	0.42	1.0–500.0
Q-3-*O*-^2^″RhaGlc (**24**)	*y* = 0.016·*x* – 0.015	0.9998	1.32·10^−3^	0.29	0.88	1.0–500.0
Q-3-*O*-^4^″RhaGlc (**25**)	*y* = 0.017·*x* + 0.032	0.9998	4.91·10^−3^	0.51	1.55	2.5–500.0
Q-3-*O*-^6^″RhaGlc (**38**)	*y* = 0.019·*x* – 0.026	0.9999	3.53·10^−3^	0.45	1.36	2.5–500.0
Q-3-*O*-Glc (**40**)	*y* = 0.023·*x* + 0.050	0.9999	1.14·10^−3^	0.75	2.27	2.5–500.0
Q-3-*O*-Gal (**42**)	*y* = 0.024·*x* – 0.016	0.9999	2.28·10^−3^	0.47	1.42	2.5–500.0
3,4-Di-*O*-CQA (**44**)	*y* = 0.061·*x* – 0.011	0.9999	2.64·10^−3^	0.79	2.39	2.5–500.0
K-3-*O*-^6^″RhaGlc (**45**)	*y* = 0.022·*x* + 0.005	0.9999	2.08·10^−3^	0.62	1.88	2.5–500.0
3,5-Di-*O*-CQA (**50**)	*y* = 0.063·*x* – 0.046	0.9999	9.20·10^−3^	0.66	2.00	2.5–500.0
Ir-3-*O*-Glc (**54**)	*y* = 0.034·*x* – 0.020	0.9998	5.29·10^−3^	0.87	2.64	2.5–500.0
4,5-Di-*O*-CQA (**58**)	*y* = 0.062·*x* – 0.026	0.9999	6.61·10^−3^	0.83	2.51	2.5–500.0
Q (**78**)	*y* = 0.032·*x* + 0.034	0.9999	8.12·10^−3^	0.79	2.39	2.5–500.0

*A, apigenin; CQA, caffeoylquinic acid; Glc, glucose; Ir, isorhamnetin; K, kaempferol; Q, quercetin; Rha, rhamnose; ^2^″RhaGlc, 2″-rhamnosyl-glycose (neohesperidose); ^4^″RhaGlc, 4″-rhamnosyl-glycose; ^6^″RhaGlc, 6″-rhamnosyl-glycose (rutinose)*.

Concentration linear ranges from 1 to 500 μg/mL were appropriate for quantification and LOQ and LOD parameters and were 0.12–0.87 and 0.36–2.64 μg/mL, respectively. The values of intra- and inter-day precision, repeatability and stability were determined for all compounds analyzed, and their means of RSD did not exceed 3% (Table 4). The range of variation of the recovery parameter was 98.06–102.11%, and is acceptable for quantitative HPLC assay according the guidelines established by the International Conference on the Harmonization of Technical Requirements for the Registration of Pharmaceuticals for Human Use (2005).

**Table 4:** Intra- and inter-day precision, repeatability, stability and recovery for 15 compounds.

**Compound**	**Precision intra-day (RSD%)**	**Precision inter-day (RSD%)**	**Repeatability (RSD%)**	**Stability (RSD%)**	**Recovery (%)**
	***n* = 5**	***n* = 4**	***n* = 7**	***n* = 7**	***n* = 5**
4-*O*-CQA (**1**)	1.82	2.14	1.27	1.37	100.52
5-*O*-CQA (**10**)	0.91	1.22	1.02	1.14	100.06
1,3-Di-*O*-CQA (**14**)	1.54	2.39	1.53	1.17	101.18
A-6-*C*-Glc-4″-*O*-Glc (**19**)	1.62	1.89	1.78	1.67	98.15
Q-3-*O*-^2^″RhaGlc (**24**)	2.04	2.57	2.18	2.14	98.06
Q-3-*O*-^4^″RhaGlc (**25**)	2.17	2.94	2.63	2.10	98.52
Q-3-*O*-^6^″RhaGlc (**38**)	1.50	1.97	1.39	1.22	100.27
Q-3-*O*-Glc (**40**)	0.98	1.32	1.27	1.35	99.25
Q-3-*O*-Gal (**42**)	1.07	1.29	1.40	1.27	99.74
3,4-Di-*O*-CQA (**44**)	1.39	2.04	1.62	1.29	99.63
K-3-*O*-^6^″RhaGlc (**45**)	1.25	1.67	1.35	1.30	98.07
3,5-Di-*O*-CQA (**50**)	1.17	1.93	1.27	1.07	102.11
Ir-3-*O*-Glc (**54**)	1.67	2.19	1.92	1.35	99.32
4,5-Di-*O*-CQA (**58**)	0.93	1.67	1.35	1.35	101.37
Q (**78**)	0.83	1.35	1.09	0.92	98.14

*A, apigenin; CQA, caffeoylquinic acid; Glc, glucose; Ir, isorhamnetin; K, kaempferol; Q, quercetin; Rha, rhamnose; ^2^″RhaGlc, 2″-rhamnosyl-glycose (neohesperidose); ^4^″RhaGlc, 4″-rhamnosyl-glycose; ^6^″RhaGlc, 6″-rhamnosyl-glycose (rutinose)*.

### Chemical comparison of 12 *Artemisia* species based on content of 15 phenolic compounds and principal component analysis (PCA)

The quantification of 15 phenolic principal compounds in 12 *Artemisia* extracts is presented in Table 5. The content of 4-*O*-caffeoylquinic acid (**1**) in the extracts analyzed ranged from trace amounts (*A. anethifolia*) to 5.64 mg/g (*A. umbrosa*). Another monosubstituted phenylpropanoid, 5-*O*-caffeoylquinic acid (**10**), dominated in the extracts of *A. anethifolia* (78.88 mg/g), *A. sericea* (20.28 mg/g) and *A. tanacetifolia* (73.71 mg/g). The highest content of **10** was noticed for *A. commutata* (127.99 mg/g) extract and the lowest value was found in *A. desertorum* extract (21.24 mg/g).

Table 5Content of 15 phenolic compounds in 12 *Artemisia* extracts (mg/g dry weight ± SD).

**Table:** 

***Artemisia* extract**	**Caffeoylquinic acids**						
	**1**	**10**	**14**	**44**	**50**	**58**	**Total**
*A. anethifolia*	<0.01	78.88 ± 1.57	<0.01	<0.01	70.76 ± 1.41	6.23 ± 0.12	155.87
*A. commutata*	3.14 ± 0.06	127.99 ± 2.55	3.75 ± 0.05	4.28 ± 0.08	243.61 ± 4.87	<0.01	382.77
*A. desertorum*	4.74 ± 0.08	21.24 ± 0.42	1.00 ± 0.02	1.24 ± 0.02	60.51 ± 1.21	3.75 ± 0.07	92.48
*A. integrifolia*	1.58 ± 0.03	63.73 ± 1.27	0.87 ± 0.02	4.32 ± 0.08	87.02 ± 1.74	22.71 ± 0.45	180.23
*A. latifolia*	0.61 ± 0.01	35.19 ± 0.70	1.21 ± 0.02	<0.01	58.55 ± 1.17	8.82 ± 0.17	104.38
*A. leucophylla*	2.57 ± 0.05	48.89 ± 0.98	1.23 ± 0.02	5.35 ± 0.10	108.18 ± 2.16	16.70 ± 0.33	182.92
*A. macrocephala*	0.81 ± 0.02	47.15 ± 0.94	6.74 ± 0.14	19.83 ± 0.39	13.42 ± 0.26	7.66 ± 0.15	95.61
*A. messerschmidtiana*	4.14 ± 0.08	53.06 ± 1.06	1.09 ± 0.02	0.82 ± 0.02	125.76 ± 2.51	12.98 ± 0.25	197.85
*A. palustris*	0.98 ± 0.02	26.59 ± 0.53	1.76 ± 0.03	2.77 ± 0.05	7.35 ± 0.14	3.23 ± 0.06	42.68
*A. sericea*	1.02 ± 0.02	20.28 ± 0.41	1.19 ± 0.02	<0.01	10.67 ± 0.21	<0.01	33.16
*A. tanacetifolia*	3.46 ± 0.06	73.71 ± 1.47	1.49 ± 0.03	4.67 ± 0.09	65.17 ± 1.29	1.06 ± 0.02	149.56
*A. umbrosa*	5.64 ± 0.11	30.05 ± 0.60	0.73 ± 0.01	5.61 ± 0.11	57.74 ± 1.15	10.86 ± 0.21	110.63

**Table:** 

	**FLAVONOIDS**				
***Artemisia*** **extract**	**19**	**24**	**25**	**38**	**40**
*A. anethifolia*	1.96 ± 0.03	0.00	0.00	9.52 ± 0.19	8.81 ± 0.17
*A. commutata*	8.40 ± 0.16	8.48 ± 0.16	<0.01	8.73 ± 0.18	1.02 ± 0.02
*A. desertorum*	0.00	<0.01	<0.01	1.61 ± 0.03	<0.01
*A. integrifolia*	0.71 ± 0.01	0.00	0.00	<0.01	9.73 ± 0.19
*A. latifolia*	0.00	0.00	0.00	11.35 ±0.22	15.75 ± 0.28
*A. leucophylla*	0.00	<0.01	<0.01	6.01 ± 0.12	5.89 ± 0.12
*A. macrocephala*	0.00	0.00	0.00	<0.01	42.17 ± 0.75
*A. messerschmidtiana*	0.00	<0.01	<0.01	2.28 ± 0.04	5.87 ± 0.12
*A. palustris*	0.00	5.90 ± 0.11	16.14 ± 0.32	<0.01	82.31 ± 1.65
*A. sericea*	0.00	0.00	0.00	<0.01	0.00
*A. tanacetifolia*	0.00	0.00	0.00	0.41 ± 0.01	1.26 ± 0.03
*A. umbrosa*	0.00	4.08 ± 0.08	2.82 ± 0.05	7.55 ± 0.15	3.25 ± 0.06
***Artemisia*** **extract**	**42**	**45**	**54**	**78**	**Total**
*A. anethifolia*	<0.01	<0.01	<0.01	0.00	20.29
*A. commutata*	2.49 ± 0.05	<0.01	<0.01	<0.01	29.12
*A. desertorum*	<0.01	0.00	<0.01	0.00	1.61
*A. integrifolia*	<0.01	<0.01	<0.01	0.00	10.44
*A. latifolia*	0.00	14.70 ± 0.27	0.00	0.00	41.80
*A. leucophylla*	0.00	<0.01	<0.01	<0.01	11.90
*A. macrocephala*	0.00	0.00	7.66 ± 0.15	1.76 ± 0.04	51.59
*A. messerschmidtiana*	0.00	<0.01	0.00	0.00	8.15
*A. palustris*	<0.01	17.97 ± 0.35	12.82 ± 0.25	<0.01	135.14
*A. sericea*	0.00	0.00	0.00	0.00	<0.01
*A. tanacetifolia*	2.00 ± 0.04	0.00	0.44 ± 0.01	0.00	4.11
*A. umbrosa*	<0.01	<0.01	<0.01	0.00	17.70

*Caffeoylquinic acids: **1**, 4-O-caffeoylquinic acid; **10**, 5-O-caffeoylquinic acid; **14**, 1,3-di-O-caffeoylquinic acid; **44**, 3,4-di-O-caffeoylquinic acid; **50**, 3,5-di-O-caffeoylquinic acid; **58**, 4,5-di-O-caffeoylquinic acid. Flavonoids: **19**, apigenin-6-C-Glc-4″-O-Glc (isosaponarin); **24**, quercetin-3-O-^2^″RhaGlc (calendoflavobioside); **25**, quercetin-3-O-^4^″RhaGlc; **38**, quercetin-3-O-^6^″RhaGlc (rutin); **40**, quercetin-3-O-Glc (isoquercitrin); **42**, quercetin-3-O-Gal (hyperoside); **45**, kaempferol-3-O-6″RhaGlc (nicotiflorin); **54**, isorhamnetin-3-O-Glc; **78**, quercetin*.

Disubstituted phenylpropanoids were represented by the minor compound 1,3-di-*O*-caffeoylquinic acid (**14**) with the maximum content revealed in *A. macrocephala* extract (6.74 mg/g) and traces for *A. anethifolia* extract; 3,4-di-*O*-caffeoylquinic acid (**44**) also had a maximal value for *A. macrocephala* extract (19.83 mg/g) and traces for *A. anethifolia, A. latifolia* and *A. sericea* extracts. Disubstituted 3,5-di-*O*-caffeoylquinic acid (**50**) was the predominant compound for a number of *Artemisia* extracts: *A. commutata* (243.61 mg/g), *A. desertorum* (60.51 mg/g), *A. integrifolia* (87.02 mg/g), *A. latifolia* (58.55 mg/g), *A. leucophylla* (108.18 mg/g), *A. messerschmidtiana* (125.76 mg/g), *A. tanacetifolia* (65.17 mg/g) and *A. umbrosa* (57.74 mg/g). The highest content of 4,5-di-*O*-caffeoylquinic acid (**58**) was noticed for the *A. integrifolia* extract (22.71 mg/g), while the traces were found for A. *commutata* and *A. sericea*. The maximum total content of phenylpropanoids was detected for *A. commutata* extract (382.77 mg/g) that significantly exceeded other total contents. The lowest total content was noticed for *A. sericea* extract (33.16 mg/g).

Principal flavonoids were presented mostly by flavonol glycosides (**24**, **25**, **38**, **40**, **42**, **45**, **54**), aglycone (**78**) and flavone glycoside (**19**). Isosaponarin (**19**), the derivative of apigenin, was quite rare compound that was quantified only in the extracts of *A. anethifolia* (1.96 mg/g), *A. commutata* (8.40 mg/g) and *A. integrifolia* (0.71 mg/g). The majority of flavonol glycosides were quercetin derivatives (**24**, **25**, **38**, **40**, **42**). Calendoflavobioside (**24**) also was analyzed in some *Artemisia* extracts studied: in the extracts of *A. commutata* (8.48 mg/g), *A. palustris* (5.90 mg/g) and *A. umbrosa* (4.08 mg/g). The highest content of quercetin-3-*O*-(4″-*O*-rhamnosyl)glycoside (**25**) was noticed for *A. palustris* extract (16.14 mg/g), while the remaining species were characterized by minor amounts or traces of **25**. The presence of rutin (**38**) was revealed in all extracts analyzed. Isoquercitrin (**40**) was the predominant compound for *A. palustris* extract (82.31 mg/g) and was absent only in *A. sericea* extract. The presence of hyperoside was observed (**42**) in the extracts of *A. commutata* (2.49 mg/g) and *A. tanacetifolia* (2.00 mg/g). Flavonol glycoside, nicotiflorin (**45**), the derivative of kaempferol, was discovered with a maximum value in *A. palustris* extract (17.97 mg/g) and was absent in the extracts of *A. desertorum, A. macrocephala, A. sericea*, and *A. tanacetifolia*.

**Figure 6:**
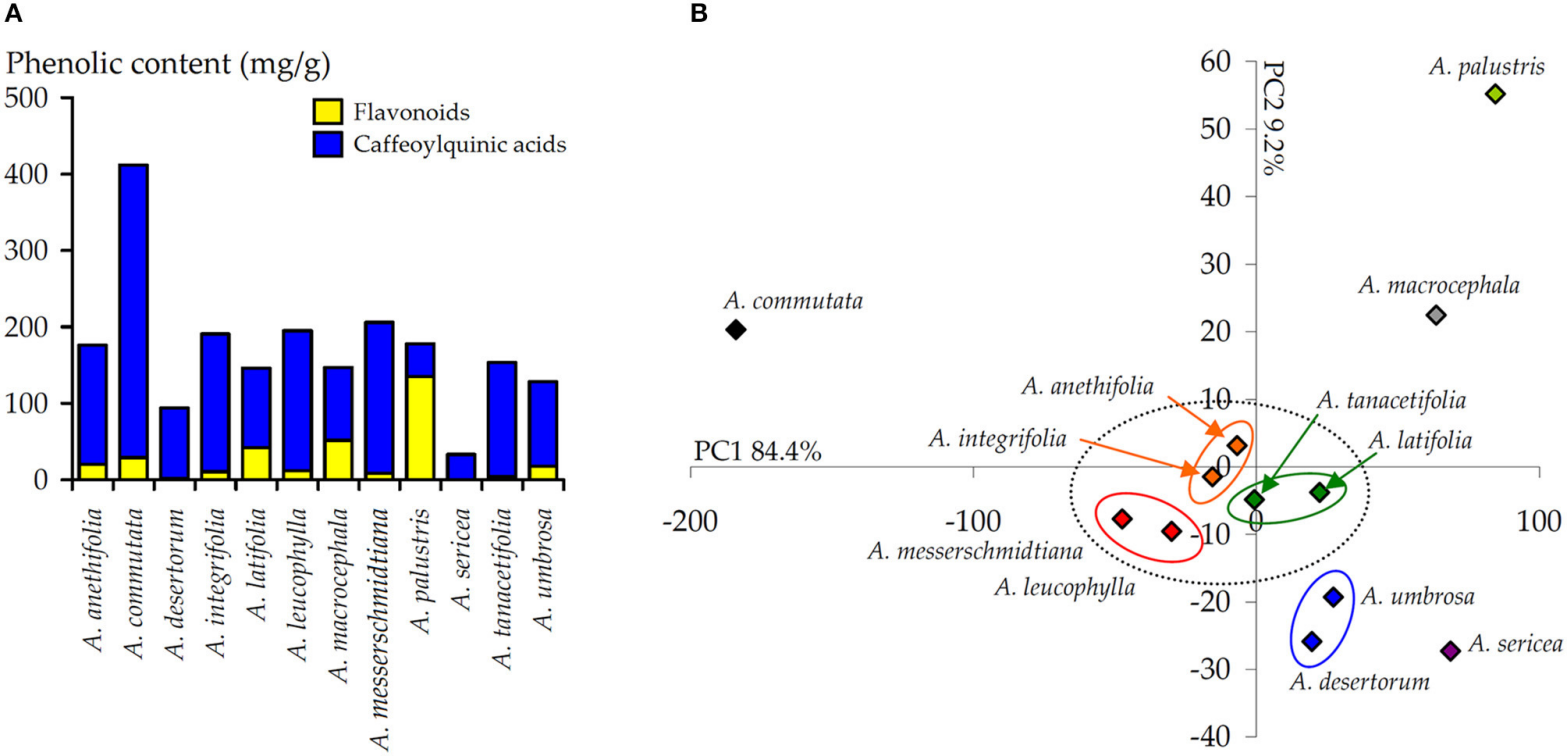
**(A)** Total content of flavonoids and CQA in the extracts of 12 *Artemisia* species. **(B)** Results of principal component analysis (PCA) used the content of 15 phenolic compounds in the extracts of 12 *Artemisia* species.

Isorhamnetin derivative, isorhamnetin-3-*O*-glucoside (**54**), also was noted with the highest content in *A. palustris* extract (12.82 mg/g). Flavonol aglycone, quercetin (**78**), was found only in *A. macrocephala* extract (1.76 mg/g) in a quantity exceeding a trace. The maximum total content of flavonoids was detected for *A. palustris* extract (135.14 mg/g) that significantly exceeded other total contents (Figure 6A). It is interesting to note that *A. sericea* extract either did not contain flavonoids, in general, or contained them in trace amounts.

The obtained data on the content of 15 phenolic compounds in the extracts of 12 *Artemisia* species were subjected to principal component analysis (PCA) (Figure 6B). The final scores plot of PCA, as a two-component model, cumulatively consider 93.6% of total variables (PC1, 84.4%; PC2, 9.2%). In the space of the main axes of the ordination, four groups were formed, while the largest number of the samples was located at the center of the diagram. The main part of this group consisted of three pairs elements as *A. anethifolia*-*A. integrifolia, A. tanacetifolia*-*A. latifolia* and *A. messerschmidtiana*-*A. leucophylla*. Also, near the centrally located group, the elements of another pair were located *A. umbrosa*-*A. desertorum*. In contrast to the main group of elements, four species (*A. commutata, A. palustris, A. sericea, A. macrocephala*) were not associated into groups.

### Inhibitory activity of pure caffeoylquinic acids against α-amylase and α-glucosidase

Because a high correlation was obtained between the caffeoylquinic acid (CQA) content in *Artemisia* extracts and extract's enzyme inhibitory activity, some pure compounds were assayed for their inhibitory potential against α-amylase and α-glucosidase. Ten compounds with various degrees of substitution of the quinic acid skeleton were divided into three groups. There were four mono-substituted CQAs (1-*O*-CQA, 3-*O*-CQA; 4-*O*-CQA; 5-*O*-CQA), five di-substituted CQAs (1,3-di-*O*-CQA; 1,5-di-*O*-CQA; 3,4-di-*O*-CQA; 3,5-di-*O*-CQA; 4,5-di-*O*-CQA) and one tri-substituted CQA (3,4,5-tri-*O*-CQA). The inhibitory effect of the tested CQAs is summarized in Table 6.

**Table 6:** Inhibitory activity of pure caffeoylquinic acids (CQA) against α-amylase and α-glucosidase (IC_50_, μM).

**Substance**	**Inhibitory activity against enzyme as IC**_**50**_	
	**α-Amylase (μM)**	**α-Glucosidase (μM)**
1-*O*-CQA^*^	172.47 ± 5.34^h^	1240.35 ± 42.10^f^
3-*O*-CQA	169.50 ± 4.41^h^	1209.63 ± 41.12^f^
4-*O*-CQA	109.59 ± 3.39^g^	1028.32 ± 40.01^ef^
5-*O*-CQA	84.92 ± 2.37^ef^	713.88 ± 19.98^cd^
1,3-Di-*O*-CQA	100.67 ± 2.81^fg^	983.53 ± 28.52^de^
1,5-Di-*O*-CQA	77.43 ± 2.24^de^	753.92 ± 23.37^c^
3,4-Di-*O*-CQA	67.17 ± 2.01^cd^	209.72 ± 6.50^b^
3,5-Di-*O*-CQA	51.29 ± 1.64^bc^	184.34 ± 5.34^b^
4,5-Di-*O*-CQA	42.32 ± 1.18^ab^	62.14 ± 1.49^a^
3,4,5-Tri-*O*-CQA^*^	40.57 ± 1.05^a^	61.08 ± 1.77^a^
Acarbose	482.54 ± 15.44^i^	1875.33 ± 60.01^g^

Substances marked with asterisks (^*^) were not detected in Artemisia species studied. Mean values ± standard deviations. Statistical analysis was performed by one-way ANOVA (Tukey test). In each column, different letters (a–g) stand for significant statistical different data (p < 0.05).

The trisubstituted caffeoylated analog of quinic acid, 3,4,5-tri-*O*-CQA, as well as 4,5-di-*O*-CQA, exhibited stronger activity as α-amylase inhibitors with IC_50_ values of 40.57 and 42.32 μM, respectively, than acarbose (IC_50_ = 482.54 μM). Two disubstituted CQAs, 3,5-di-*O*-CQA and 3,4-di-*O*-CQA, demonstrated similar activities, with IC_50_ = 51.29 and 67.17 μM, respectively. The other di-CQAs and all mono-substituted CQAs showed moderate inhibition of α-amylase in the range of IC_50_ from 77.43 (1,5-di-*O*-CQA) to 172.47 μM (1-*O*-CQA).

For the α-glucosidase inhibition, the general trend of activity was similar. Two compounds, 3,4,5-tri-*O*-CQA and 4,5-di-*O*-CQA, were the most active (IC_50_ = 61.08 and 62.14 μM, respectively) greatly exceeding the activity of the reference inhibitor acarbose (IC_50_ = 1875.33 μM). The additional di-CQAs showed average activities with IC_50_ range 184.34–983.53 μM. The potency of mono-substituted CQAs against α-glucosidase was close to acarbose, because their IC_50_ values were from 713.88 (5-*O*-CQA) to 1240.35 μM (1-*O*-CQA).

## Discussion

Some studies have shown that plant species of the *Artemisia* genus, used in the ethnomedical systems of Asia, have antidiabetic properties (Dabe and Kefale, 2017). The people living in the territory of present-day Northern Asia or Siberia, widely used *Artemisia* extracts for the treatment of diabetes-like conditions. To our knowledge, this is the first report that validates the carbohydrate digestive enzyme inhibiting activity of *Artemisia* species, popular in Siberia, using ethnopharmacological information. Additionally, we identified the compounds responsible for the biological activity. Twelve *Artemisia* species selected for the investigation were characterized as concentrators and superconcentrators of flavonoids with quantitative content ranging from 2.46 (*A. desertorum*) to 202.67 mg/g (*A. palustris*). A similar conclusion was reached by quantification of caffeoylquinic acids (CQAs) found ranging from 26.46 (*A. sericea*) to 514.65 mg/g (*A. commutata*). All studied *Artemisia* extracts were effective inhibitors of α-amylase (IC_50_ = 150.24–384.14 μg/mL; acarbose IC_50_ = 311.24 μg/mL) and α-glucosidase (IC_50_ = 214.42–754.12 μg/mL; acarbose IC_50_ = 1209.59 μg/mL), two digestive enzymes involved in the process of increasing postprandial blood glucose level (Yin et al., 2014). Correlation analysis revealed a direct relationship between the content of CQAs in *Artemisia* extracts and their enzyme inhibitory activity, in contrast to flavonoids that showed weak correlation links. Removal of the phenolic compounds from *Artemisia* extracts resulted in a complete loss of inhibitory properties (IC_50_ > 2,000 μg/mL) and indicated the leading role of these natural compounds in the manifestation of the hypoglycemic effect. Thus, we can confirm the appropriateness of the application of Siberian *Artemisia* species as α-amylase and α-glucosidase inhibitors, while identifying the phenolic compounds, and especially CQAs, as the main active compounds.

To establish the metabolomic profile of the phenolic compounds, a detailed chromatographic investigation of all *Artemisia* extracts was performed. At the preliminary stage of the investigation of HPLC profile of the total extracts of *Artemisia*, it was found that caffeoylquinic acids (CQA), the predominant compounds, interfered with the chromatographic separation process, disguising minor flavonoid glycosides and other compounds. To address this deficiency, methanol extracts were preliminarily subjected to solid phase extraction (SPE) on polyamide, thereby removing of the major proportion of CQA to obtain the flavonoid-enriched SPE fraction. The effectiveness of that prechromatographic treatment of plant extracts to analyze minor compounds was earlier shown (Svehliková et al., 2004; Olennikov et al., 2015a,b; Kashchenko et al., 2017b). The application of two types of extracts to analyze *Artemisia* species allowed us to achieve greater specificity and sensitivity of metabolomic profiling.

As far as we are concerned, the present study is the first such large-scale scientific project on the investigation of Siberian *Artemisia* species, resulting in the presence of over a 100 compounds revealed with the capacities of the HPLC-DAD-ESI-TQ-MS/MS method in 12 *Artemisia* species. Previously, the information of any phenolic compounds was known only for *A. integrifolia* (flavonoids; Wang J.-L. et al., 2016), *A. palustris* (flavonoids; Chemesova et al., 1978) and *A. tanacetifolia* (coumarins; Szabó et al., 1985). The data on the phenolic compounds of *A. anethifolia, A. latifolia, A. macrocephala*, and *A. umbrosa* were collected for the first time, and our research is the first to have studied the following five species: *A. commutata, A. desertorum, A. leucophylla, A. messerschmidtiana* and *A. sericea*.

Phenylpropanoid quinates were the most common group of the phenolic compounds found in all types of *Artemisia*, including mono- and dicaffeoylquinic acids with different types of substitutions, as well as 5-*O*-coumaroylquinic acid, (**22**) found only in *A. latifolia* and *A. tanacetifolia*. Previously it has been repeatedly shown that monotypic and mixed quinates of cinnamic, caffeic and ferulic acids are obligatory components of *Artemisia* species, including *A. absinthium* (Fiamegos et al., 2011), *A. annua* (Han et al., 2008), *A. gmelinii* (Könczöl et al., 2012), *A. iwayomogi* (Lee et al., 2017), *A. princeps* (Cui et al., 2009), *A. vulgaris* (Fraisse et al., 2011), and others. In contrast to phenylpropanoid quinates, there were only four species of *Artemisia* in which the representatives of a rare group of phenylpropanoid glycosides were detected, including glycosides of caffeic (**5**; *A. messerschmidtiana*), ferulic (**8**) and dehydroferulic acids (**3**; *A. anethifolia*), which, together with glycoside of protocatechuic acid (**2**; *A. sericea*), had not been described previously for the genus *Artemisia*. This applied equally to the isomers of melilotoside (**17**, **30**) from *A. tanacetifolia*, previously mentioned only for *A. splendens* (Afshar et al., 2017).

Coumarins are considered ordinary common group of compounds for the genus *Artemisia* (Al-Hazimi and Basha, 1991). These compounds were discovered in seven studied species in the present research, including 7-*O*-glycosides of umbelliferone (**4**) and scopoletin (**18**). Only in two species, *A. latifolia* and *A. tanacetifolia*, coumarin-hemiterpene esters, such as lacarol (**74**), desoxylacarol (**83**) and methyllacarol (**91**) were identified. Both species are related to the Laciniatae subsection of the Abrotanum section, that contains *A. laciniata* and *A. armeniaca*, which **74**, **83**, and **91** were identified earlier (Hofer et al., 1986; Mojarrab et al., 2011). Such a limited rate of detectability of these compounds, only within the subsection Laciniatae, indicates their considerable chemosistematic significance.

Davididenin (**85**) and its methyl esters (**98**, **105**, **112**) were detected only in *A. palustris*, which is related to the small Auratae subsection of the of the Abrotanum section, together with the unexplored Asian species *A. aurata*. Despite the lack of scientific information, in view of their uniqueness, these compounds are promising systematic markers for the species of this subsection.

The flavonoid group of flavones is widely distributed in Siberian *Artemisia* species and has been identified in all studied species. Specific accumulation of *C*,*O*-glycosides of apigenin has been established for species of the section *Artemisia* (*A. integrifolia, A. leucophylla, A. umbrosa*), as was also shown earlier for the taxonomically similar species *A. vulgaris* (Lee et al., 1998). A similar pattern of accumulation was found for two representatives of the subgenus Dracunculus – *A. commutata* (subsection Commutatae, section Campestris) and *A. desertorum* (subsection Japonica, section Dracunculus), that can concentrate the flavonoids of this group (Kaneta et al., 1978). *A. sericea* was the only species capable of concentrating flavone glycosides of various types, such as the glycosides of apigenin, luteolin, 6-hydroxyluteoline and others detected. This species relates to the subsection Frigidae (section Absinthium) together with *A. frigida*, known for its ability to biosynthesize flavones (Wang Q. et al., 2016). *A. anethifolia*, in particular, was distinguished from the other studied *Artemisia* species, which selectively accumulated chrysoeriol glycosides (**34**, **61**, **67**). Probably, this chemical feature is characteristic for the subsection Anethifoliae (section Absinthium), to which this species belongs. Flavone glycosides were not found in *A. palustris*, which contains only aglycons, indicating the uniqueness of this species and its distant taxonomic position from other species. It was clearly shown that individual flavones (and even their groups) were distributed extremely unevenly in the species studied. A completely different pattern of occurrence was found for flavonoid glycosides of the flavonol group, where a high frequency for at least eight compounds was observed. These compounds included nicotiflorin (**45**), astragalin (**53**), calendoflavobioside (**24**), quercetin-3-*O*-(4″-rhamnosyl)glucoside (**25**), rutin (**38**), isoquercitrin (**40**), narcissin (**46**) and isoramnetin-3-*O*-glucoside (**54**). There was an earlier reference to the diversity of flavonols of the genus *Artemisia* and their almost obligate presence in all species of the genus (Al-Hazimi and Basha, 1991). However, despite their low taxonomic significance, the species *A. sericea* (subsection Frigidae) should be noted to contain only traces **24** and **25**, which can also be considered as a sign of species differentiation at the level of the Absinthium section. Eriodictyol glycosides (**12**, **16**) were found only in *A. macrocephala* and can be considered a chemical feature of this species, or even the subsection Absinthium (section Absinthium), to which it is related.

In general, it is necessary to note the unusual chemodiversity of the types of the phenolic compounds we found in the Siberian species of *Artemisia* by the HPLC-DAD-ESI-TQ-MS/MS method. The aforementioned chemical features of accumulation of the individual compounds or their groups can be used further as a convenient taxonomic tool for analysis of the genus *Artemisia* as a whole. The uniqueness of the individual compound structures can be the reason for the high biological activity of the investigated plant extracts.

The investigation of the accumulation features of individual compounds in 12 *Artemisia* species showed a significant variability in their quantitative profiles, which is probably due to the taxonomic position of the species within the genus. Caffeoylquinic acids (CQA) were not only obligate components, but also the dominant group of the phenolic compounds for all studied *Artemisia* species (95.61–382.77 mg/g), except *A. palustris* where flavonoids prevailed. 5-*O*-caffeoylquinic acid (**10**) and 3,5-di-*O*-caffeoylquinic acid (**50**) were two major phenylpropanoid quinates and required special attention, owing to widely varying content within the range of 20.28–127.99 and 7.35–243.61 mg/g, respectively. Both compounds are the most frequently detectable and quantifiable pair of dominant phenylpropanoids in various types of *Artemisia*. For example, it is known that, the concentration of **50** in the ethyl acetate extract of *A. selengensis* is 106.52 mg/g with a total CQAs content of 241.46 mg/g (Li et al., 2018), the presence of **10** (4.1 mg/g) and **50** (11.99 mg/g) were identified in the methanol extract of *A. princeps* (Lee et al., 2011). For the *A. iwayomogi* herb, the presence of **10** and **50** were discovered at 1.66–33.61 and 14.33–68.87 mg/g, respectively (Lee et al., 2017). Diverse CQAs have also been detected in other Asian species of *Artemisia*, such as *A. annua* (Han et al., 2008), *A. argyi* (Zhang et al., 2012), *A. capillaris* (Seo et al., 2003), *A. gmelinii* (Könczöl et al., 2012), and *A. montana* (Jung et al., 2011). The high content of CQAs in the Siberian species of *Artemisia* indicates their promise as plant sources of phenylpropanoids. There is a high probability that CQAs are obligatory components of the *Artemisia* genus phenolome, in contrast to flavonoids in which total concentration was significantly lower in the studied species (<0.01–135.14 mg/g). According to literature, the content of flavonoids in previously studied *Artemisia* extracts was 2.35 mg/g in *A. splendens*, 2.60 mg/g in *A. spicigera* (Afshar et al., 2012), 17.21–29.68 mg/g in *A. campestris* (Khettaf et al., 2016), 18.85–87.04 mg/g in A. *absinthium* (Lee et al., 2016), 67.98 mg/g in *A. vulgaris*, 71.41 mg/g in *A. arborescens*, 93.86 mg/g in *A. campestris*, 109.02 mg/g in *A. santonicum* and 121.96 mg/g in *A. scoparia* (Baykan Erel et al., 2012). Thus, the Siberian species of *Artemisia* were close to other Eurasian representatives of the same genus, due to the ability to accumulate flavonoids.

In our early studies of natural enzyme inhibitors, high efficiency against α-amylase and α-glucosidase of the phenolic compounds of various structural types was shown, including flavonoids (Kashchenko et al., 2017a; Olennikov and Kashchenko, 2017), coumarins (Olennikov et al., 2017c), phenylpropanoids (Olennikov and Kashchenko, 2014), arylheptanoids (Olennikov and Kashchenko, 2015) and ellagitannins (Kashchenko et al., 2017b). Caffeic acid, which had the greatest biological effect in the series of homologous compounds, was of interest. The investigated *Artemisia* species were characterized by high content of caffeoylquinic acids (CQAs) derived from caffeic acid, and thereby high inhibitory activity against digestive enzymes was expected. As was shown earlier, the correlation analysis indicated an unambiguous relationship between the concentration of CQAs in the plant extract and its inhibitory activity. The research of CQAs of different structural types confirmed this assumption. Di-CQAs were the most effective inhibitors of α-amylase and α-glucosidase, which exceeded the activity of mono-CQAs, indicating the importance of the number of aromatic substituents in the structure of the compound. The location of the caffeoyl substituent influenced the activity of the compound, while the greatest inhibitory effect was established for 5-*O* and 4-*O*-substituted derivatives, in contrast to 1-*O*- and 3-*O*-derivatives. This effect was retained for di-substituted CQAs and even tri-substituted CQAs (3,4,5-tri-*O*-CQAs), although the latter compounds were not identified in the species studied. The most likely reason of this effect could be the ability of CQAs to interact with the functional groups of α-amylase and α-glucosidase that, ultimately, led to a decrease in their effective activity and ability to decompose carbohydrates. Previously, for individual CQAs, it was shown that hydroxyl groups of CQAs can bind with polar groups of enzymes (-OH, -SH, -NH), affecting their hydrophobic properties (Moorthy et al., 2012a,b). Besides, this effect can involve the secondary structure of enzymes, as was shown for 5-*O*-CQA and 3,5-di-*O*-CQA (Xu et al., 2015). Similar activity was detected for individual CQAs detected in *Tussilafo farfara* (Gao et al., 2008), *Lonicera fulvotomentosa* (Tang et al., 2008), *Coffea arabica* (Narita and Inouye, 2011), and *Ilex kudingcha* (Xu et al., 2015), but for the first time, it was revealed for compounds characteristic for Siberian *Artemisia* species.

## Conclusion

The present paper demonstrated the results of the first comprehensive investigation of the Siberian *Artemisia* species as a source of bioactive phenolics with special attention paid to their inhibitory potential against of α-glucosidase and α-amylase. Bioassay guided screening gave indisputable evidence of the pronounced activity of the extracts from the 12 ethnopharmacologically selected *Artemisia* species caused by the phenolic compounds. A total 112 phenolics of various groups were unambiguously, or tentatively, identified by HPLC-DAD-ESI-TQ-MS/MS. It was suggested that the flavonoid or coumarin patterns of *Artemisia* species are as suitable chemotaxonomic tools as other groups of plant phenolics and should be also applied. Quantification data for 15 principal phenolics helped to conclude the caffeoylquinic acids (CQAs) as major constituents, and their concentration levels were in good correlation with enzyme-inhibitory activity of plant extracts. Additional study of pure compounds showed the highest inhibitory potential among di-*O*-substituted CQAs, indicating that the caffeoyl-substitution at C-5 and C-4 of the quinic acid moiety appears to be a key factor in the α-glucosidase and α-amylase inhibition. This activity can also be a candidate for bioactivity targeting and further *in vivo* investigations are required to estimate glucose-lowering effect of *Artemisia* extracts.

## Author contributions

The first author DO and CV mainly responsible for the operation of the whole experiment and the writing of the paper. NC, NK, VN, and S-WK mainly responsible for assisting the first author in the experiment. All authors responsible for providing technical guidance and experimental materials.

### Conflict of interest statement

The authors declare that the research was conducted in the absence of any commercial or financial relationships that could be construed as a potential conflict of interest.
