# Supplementary material for: Bioactive Phenolics of the Genus Artemisia (Asteraceae): HPLC-DAD-ESI-TQ-MS/MS Profile of the Siberian Species and Their Inhibitory Potential Against α-Amylase and α-Glucosidase
Source: Front Pharmacol. 2018 Jul 12;9:756. doi: 10.3389/fphar.2018.00756 (PMC6052120; doi:10.3389/fphar.2018.00756)
Supplement: Supplementary file 1 [file Table_1.DOCX]

Supplementary Material

Bioactive Phenolics of *Artemisia* Genus (Asteraceae): HPLC-DAD-ESI-TQ-MS/MS Profile of the Siberian Species and Their Inhibitory Potential against α-Amylase and α-Glucosidase

Daniil N. Olennikov^1*^, Nina I. Kashchenko^1^, Nadezhda K. Chirikova^2^, Vyacheslav M. Nikolaev^3^, Sang-Woo Kim^4^, Cecile Vennos^5^

^1^Laboratory of Medical and Biological Research, Institute of General and Experimental Biology, Siberian Division, Russian Academy of Science, Ulan-Ude, Russia

^2^Department of Biochemistry and Biotechnology, North-Eastern Federal University, Yakutsk, Russia

^3^Department of Studying the Mechanisms of Adaptation, Scientific Center of Complex Medical Sciences, Yakutsk, Russia

^4^Department of Biological Sciences, Pusan National University, Busan 46241, Republic of Korea

^5^Regulatory and Medical Scientific Affairs, Padma AG, 1 Underfeldstrasse, Hinwil CH-8340, Switzerland

*** Correspondence:**Corresponding Author
olennikovdn@mail.ru

# Supplementary Data

**Supplementary Table S1**. Yield of *Artemisia* dephenolized extracts (% of total extract weight), total flavonoid and caffeoylquinic acids (CQAs) content (mg/g) and inhibitory activity of *Artemisia* dephenolized extracts against α-amylase and α-glucosidase (IC_50_, μg/mL)

# Supplementary Table S1

Yield of *Artemisia* dephenolized extracts (% of total extract weight), total flavonoid and caffeoylquinic acids (CQAs) content (mg/g) and inhibitory activity of *Artemisia* dephenolized extracts against α-amylase and α-glucosidase (IC_50_, μg/mL)

| ***Artemisia* extract** | **Yield (%)** | **Phenolic group content (mg/g)** | | **Inhibitory activity, IC_50_ (μg/mL)** | |
| --- | --- | --- | --- | --- | --- |
|  |  | **Flavonoids** | **CQAs** | **α-Amylase** | **α-Glucosidase** |
| *A. anethifolia* | 1.9 | 0.21 ± 0.00 | 0.14 ± 0.00 | > 2000 | > 2000 |
| *A. commutata* | 1.4 | 0.44 ± 0.00 | 3.64 ± 0.06 | > 2000 | > 2000 |
| *A. desertorum* | 2.9 | > 0.01 | > 0.01 | > 2000 | > 2000 |
| *A. integrifolia* | 1.7 | > 0.01 | > 0.01 | > 2000 | > 2000 |
| *A. latifolia* | 4.8 | 0.80 ± 0.01 | > 0.01 | > 2000 | > 2000 |
| *A. leucophylla* | 3.3 | > 0.01 | 0.85 ± 0.02 | > 2000 | > 2000 |
| *A. macrocephala* | 7.8 | 0.53 ± 0.01 | > 0.01 | > 2000 | > 2000 |
| *A. messerschmidtiana* | 9.6 | > 0.01 | 0.20 ± 0.00 | > 2000 | > 2000 |
| *A. palustris* | 4.7 | 1.02 ± 0.02 | > 0.01 | > 2000 | > 2000 |
| *A. sericea* | 4.9 | > 0.01 | > 0.01 | > 2000 | > 2000 |
| *A. tanacetifolia* | 6.1 | > 0.01 | 0.56 ± 0.01 | > 2000 | > 2000 |
| *A. umbrosa* | 2.7 | > 0.01 | > 0.01 | > 2000 | > 2000 |
| Acarbose | – | – | – | 311.24 ± 8.09 | 1209.59 ± 7.02 |

Mean values ± standard deviations.
